# Impacts of *DROSHA* (rs10719) and *DICER* (rs3742330) Variants on Breast Cancer Risk and Their Distribution in Blood and Tissue Samples of Egyptian Patients

**DOI:** 10.3390/cimb46090602

**Published:** 2024-09-12

**Authors:** Aly A. M. Shaalan, Essam Al Ageeli, Shahad W. Kattan, Amany I. Almars, Nouf A. Babteen, Abdulmajeed A. A. Sindi, Eman A. Toraih, Manal S. Fawzy, Marwa Hussein Mohamed

**Affiliations:** 1 Department of Anatomy, Faculty of Medicine, Jazan University, Jazan 45142, Saudi Arabia; ashaalan@jazanu.edu.sa; 2 Department of Histology and Cell Biology, Faculty of Medicine, Suez Canal University, Ismailia 41522, Egypt; 3 Department of Basic Medical Sciences, Faculty of Medicine, Jazan University, Jazan 45141, Saudi Arabia; ealageeli@jazanu.edu.sa; 4 Department of Medical Laboratory, College of Applied Medical Sciences, Taibah University, Yanbu 46423, Saudi Arabia; skattan@taibahu.edu.sa; 5 Department of Medical Laboratory Sciences, Faculty of Applied Medical Sciences, King Abdulaziz University, Jeddah 21589, Saudi Arabia; aialmars@kau.edu.sa; 6 Hematology Research Unit, King Fahd Medical Research Center, King Abdulaziz University, Jeddah 21589, Saudi Arabia; 7 Department of Biochemistry, College of Science, University of Jeddah, Jeddah 80203, Saudi Arabia; nababteen@uj.edu.sa; 8 Department of Basic Medical Sciences, Faculty of Applied Medical Sciences, Al-Baha University, Al-Baha 65779, Saudi Arabia; asindi@bu.edu.sa; 9 Department of Surgery, School of Medicine, Tulane University, New Orleans, LA 70112, USA; 10 Genetics Unit, Department of Histology and Cell Biology, Faculty of Medicine, Suez Canal University, Ismailia 41522, Egypt; 11 Department of Biochemistry, Faculty of Medicine, Northern Border University, Arar 91341, Saudi Arabia; 12 Center for Health Research, Northern Border University, Arar 91431, Saudi Arabia; 13 Department of Medical Biochemistry and Molecular Biology, Faculty of Medicine, Suez Canal University, Ismailia 41522, Egypt; marwa_elsheikh@med.suez.edu.eg

**Keywords:** breast cancer, microRNA, *DROSHA*, *DICER*, polymorphism, susceptibility

## Abstract

MicroRNAs (miRNAs) are small, noncoding RNAs that regulate gene expression and play critical roles in tumorigenesis. Genetic variants in miRNA processing genes, *DROSHA* and *DICER*, have been implicated in cancer susceptibility and progression in various populations. However, their role in Egyptian patients with breast cancer (BC) remains unexplored. This study aims to investigate the association of *DROSHA* rs10719 and *DICER* rs3742330 polymorphisms with BC risk and clinical outcomes. This case–control study included 209 BC patients and 106 healthy controls. Genotyping was performed using TaqMan assays in blood, tumor tissue, and adjacent non-cancerous tissue samples. Associations were analyzed using logistic regression and Fisher’s exact test. The *DROSHA* rs10719 AA genotype was associated with a 3.2-fold increased risk (95%CI = 1.23–9.36, *p* < 0.001), and the *DICER* rs3742330 GG genotype was associated with a 3.51-fold increased risk (95%CI = 1.5–8.25, *p* = 0.001) of BC. Minor allele frequencies were 0.42 for rs10719 A and 0.37 for rs3742330 G alleles. The risk alleles were significantly more prevalent in tumor tissue than adjacent normal tissue (rs10719 A: 40.8% vs. 0%; rs3742330 G: 42.7% vs. 0%; *p* < 0.001). However, no significant associations were observed with clinicopathological features or survival outcomes over a median follow-up of 17 months. In conclusion, *DROSHA* rs10719 and *DICER* rs3742330 polymorphisms are associated with increased BC risk and more prevalent in tumor tissue among our cohort, suggesting a potential role in miRNA dysregulation during breast tumorigenesis. These findings highlight the importance of miRNA processing gene variants in BC susceptibility and warrant further validation in larger cohorts and different ethnic populations.

## 1. Introduction

Breast cancer (BC) represents a significant global health challenge and stands as one of the foremost causes of cancer-related deaths among women globally [1]. In 2022, it was estimated that there were around 2.308 million cases of BC, making it the most common cancer diagnosed worldwide [2]. Additionally, BC accounted for nearly 665,684 deaths in 2022, underscoring its significant contribution to morbidity and mortality among women [2]. The high incidence rate, along with the associated mortality, reinforces the necessity for ongoing research into risk factors and genetic predispositions related to BC.

Despite advances in diagnosis and treatment, the molecular mechanisms underlying BC development/progression remain incompletely understood [2]. Identifying novel genetic and epigenetic markers associated with BC risk and outcomes is essential for improving preventive measurements, early detection, and personalized therapeutic strategies [3,4].

MicroRNAs (miRNAs) are a family of “small noncoding RNAs” that modulate gene expression after transcription and are crucial for a variety of biological processes, such as cell proliferation/differentiation and apoptosis [5]. Aberrant miRNA expression has been involved in the pathogenesis of several cancers, including BC [6,7]. The biogenesis of miRNAs is a multi-step process involving several enzymes, with the ribonucleases DROSHA and DICER being two critical components [8]. DROSHA, as part of the microprocessor complex, cleaves the primary miRNA transcript into precursor miRNA in the nucleus. DICER subsequently converts the precursor miRNA into a double-stranded molecule that includes the mature miRNA guide strand and the passenger strand in the cytoplasm [9].

The dysregulated expression of DROSHA and DICER has emerged as a critical factor in BC pathogenesis [10], and their altered expression levels have been reported to impact several stages of BC development and progression [11]. This dysregulation can affect crucial cellular processes such as cell growth, invasion, metastasis, and apoptosis, all contributing to the BC malignant phenotype [12]. The intricate interplay between DICER, DROSHA, and miRNAs underscores their significance in BC biology, highlighting them as potential diagnostic and therapeutic targets for managing this disorder [13].

Genetic variants in the *DROSHA* (Gene ID: 29102) and *DICER* (Gene ID: 23405) have been investigated for their potential influence on miRNA processing and cancer susceptibility [14]. “Single-nucleotide polymorphisms (SNPs)” in these genes, such as rs10719 in *DROSHA* and rs3742330 in *DICER*, have been associated with altered risk and/or survival outcomes in several malignancies, including esophageal, ovarian, and colorectal cancers [15]. However, these SNPs’ role in BC remains poorly characterized, with limited and inconsistent evidence [16,17].

To address this gap, we conducted a case–control study to comprehensively unravel the association of *DROSHA* rs10719 and *DICER* rs3742330 polymorphisms with BC risk and clinicopathological characteristics in a sample of Egyptian women. We genotyped both SNPs in paired breast tumor and adjacent non-cancerous tissue samples, as well as in peripheral blood samples from patients with BC and healthy controls. To the best of our knowledge, this is the first study to investigate these SNPs in a BC population and compare the genotype distributions between tumor tissues and matched normal tissues.

Our findings provide new insights into the potential contributions of miRNA processing gene variants to BC susceptibility and clinical outcomes. Understanding these associations could help to identify novel biomarkers for BC risk assessment, ultimately enabling more precise and effective management of this heterogeneous disease.

## 2. Materials and Methods

### 2.1. Study Variant Selection, In Silico Analysis, and Literature Review

*DROSHA* and *DICER* genes’ genomic structures and variants were obtained from the “Ensembl Genomic database (www.ensembl.org)”. After reviewing and sorting the list, the prevalent biallelic variants, *DROSHA* rs10719 (A/G) and *DICER* rs3742330 (A/G), were identified for further study. The potential regulatory roles of these variants, influenced by their spatial genomic structures and their chromatin loop-mediated interactions with other genes and variants, were sourced from the 3DSNP database and depicted through three-dimensional visualizations showcasing gene interactions, regulatory enhancers, promoters, transcription factors, and conservation metrics (https://omic.tech/3dsnpv2/) [18]. The relevant literature was collated from the “GeneCards human gene database (www.genecards.org)” and the “National Center for Biotechnology Information (NCBI) (https://www.ncbi.nlm.nih.gov/)”, with all referenced databases last accessed on 30 March 2024.

To ensure a comprehensive understanding of the relationship between *DROSHA* rs10719 and *DICER* rs3742330 variants and their implications in various cancers, we conducted a systematic literature review. This review aimed to identify, analyze, and synthesize existing research on the associations of these variants with BC and other malignancies. The literature search used electronic databases, including “PubMed, Scopus, and Web of Science”. Relevant articles were selected based on the following criteria: studies that investigated “DROSHA rs10719” and/or “DICER rs3742330” in the context of cancer, published in peer-reviewed journals, and involving human subjects. The search strategy utilized keywords such as “DROSHA”, “DICER”, “rs10719”, “rs3742330”, and “cancer risk” in various combinations.

### 2.2. Study Population

A total of 209 female patients with BC were enrolled in the current study after obtaining the ethical approval of the institutional ethical committee (# 5027, 29 September 2022). These included (1) 103 archived paired tumor and adjacent non-tumor formalin-fixed paraffin-embedded (FFPE) tissue samples recruited from the pathology laboratory of Suez Canal University, Ismailia, and AlByan Laboratory in Port Said, Egypt, as well as (2) 106 blood samples of patients with BC obtained at the time of surgery. They had no prior history of radiotherapy or chemotherapy before tumor resection. Another 106 age-matched control blood samples were retrieved from the blood bank. Written consent was obtained from participants before they took part in this study.

### 2.3. Pathological and Clinical Assessment

A pathologist performed a post-operative pathological assessment of BC tissue specimens to determine the histopathological type, tumor size, grade, and lymph node infiltration. The Elston and Ellis modification of the Scarff–Bloom–Richardson grading system was used to grade the cancer cells, which assigns scores ranging from 1 to 3 to three parameters: tubule formation, nuclear pleomorphism, and mitotic index. Scores of 3–5 indicate well-differentiated cancer cells (grade 1), scores of 6–7 indicate moderately differentiated cancer cells (grade 2), and scores of 8–9 indicate poor or undifferentiated cancer cells (grade 3). Clinical staging was classified according to the “International Union Against Cancer (UICC)” and the “American Joint Committee on Cancer (AJCC) tumor-lymph node-metastasis (TNM)” staging system. Immunohistochemistry analysis was used to evaluate the hormone receptor status “(estrogen receptor (ER), progesterone receptor (PR), and human epidermal growth factor receptor 2 (HER2/neu))” of the tumor tissues. Patients were then classified into four molecular tumor subtypes based on the results of the immunohistochemical analysis: “(1) Luminal A: ER+, PR+, HER2−, (2) Luminal B: ER+, PR+, HER2+, (3) HER2+ subset: ER−, PR−, HER2+, and (4) Basal-like (triple negative): ER−, PR−, HER2−”.

An evaluation of the prognosis of each patient was conducted using the “Nottingham Prognostic Index (NPI)” and “Immunohistochemical Prognostic Index (IHPI)” [19]. The NPI was based on three prognostic factors: (i) tumor size, (ii) histological grade, and (iii) lymph node status; it was grouped into three prognostic classes according to the NPI results—good, moderate, and poor. The IHPI scoring system complemented the NPI and was based on the HER2, ER, and PR statuses. Patients were then ranked from 0 to 4 points and divided into three classes—good, moderate, and poor. In addition, the “European Society of Medical Oncology (ESMO)” clinical recommendations for the follow-up of primary BC was used to predict the risk of recurrence and divided into three categories—low, intermediate, and high [20]. The follow-up of the patients was conducted to assess loco-regional recurrence, disease-free survival, and overall survival.

### 2.4. Sample Collection

FFPE tissue and blood samples (3 mL) on EDTA-coated vacutainers were collected from patients with BC. FFPE tissue samples included both tumor tissue and adjacent non-tumor tissue. Blood samples were obtained at the time of surgery prior to any treatment. Control blood samples were age-matched to the cases and retrieved from the blood bank.

### 2.5. Allelic Discrimination Analysis

After genomic DNA isolation using a commercially available DNA extraction kit, “QIAamp DNA Blood Mini kit (Cat. No. 51104, QIAGEN, Hilden, Germany)”, following the procedures outlined by the supplier’s guidelines, measurements to assess the quality/quantity of the isolated genetic material were conducted through “Nanodrop-1000 spectrophotometer (NanoDrop Tech., Wilmington, NC, USA)”. The DNA samples were then carefully preserved at −80 degrees Celsius for subsequent allelic discrimination polymerase chain reaction (PCR) analysis. The rs10719 in DROSHA and rs3742330 in DICER were genotyped using TaqMan SNP genotyping assays on the “StepOne™ Real-Time PCR system” (Applied Biosystems, Foster City, CA, USA). The assays C___7761648_10 and C__27475447_10 with the catalog numbers 4351379 and 4351379, respectively (Applied Biosystems), probed the wild/mutant alleles in the following context sequences: “[VIC/FAM]TATTTTATTTCAATGAGCACACTTC[A/G]TTCATTGTCTGCAGGAAAC AGGC and CTTCAATCTTGTGTAAAGGGATTAG[A/G]CACCCTAACAGAGCAAGA TCCAATA”, respectively; these sequences aligned with the reference genome build GRCh38. The exact formulations and the concentrations of the reagents used in each PCR have been described in prior studies [21]. PCR was accomplished in a 25 μL volume mixture containing 1× TaqMan Genotyping Master Mix, 1× SNP genotyping assay mix, and 20 ng genomic DNA. The PCR cycling protocol consisted of an initial step at 95 °C for 10 min, followed by 40 cycles comprising 15 s at 95 °C and 1 min at 60 °C [22]. To mitigate the risk of contamination, no-template controls accompanied each batch of experiments, and to ensure reliability, a subset of the samples, amounting to 10%, was subsequently retested, achieving total agreement with the initial results. The post-amplification analysis was executed using specialized software provided by the PCR system’s manufacturer.

### 2.6. Statistical Analysis

SNP analysis, including the Hardy–Weinberg equilibrium, allele, and genotype frequencies, was performed using SNPStat (www.snpstats.net). The Chi-square test was used for comparison. Adjusted odds ratios (ORs) with 95% confidence intervals (CI) were calculated using logistic regression models for multiple genetic association models, adjusting for relevant covariates [21]. The association of the SNPs with clinical and pathological markers was assessed using Fisher’s exact test for categorical variables and Student’s *t*-test for continuous variables. A paired *t*-test was applied to compare genotypes between paired tumor and non-tumor tissue samples. Univariate and multivariate logistic regression analyses were performed to estimate the impacts of SNPs on BC risk, calculating ORs and 95% CIs. Survival analyses were not conducted as preliminary assessments yielded insignificant associations between the studied variants and survival outcomes. All statistical analyses were conducted with a two-sided approach, and a *p*-value of less than 0.05 was deemed statistically significant. SPSS software version 27.0 (IBM Corporation, Armonk, NY, USA) was applied for statistical analysis.

## 3. Results

### 3.1. Selection of Single-Nucleotide Polymorphisms and In Silico Analysis

**Figure 1:**
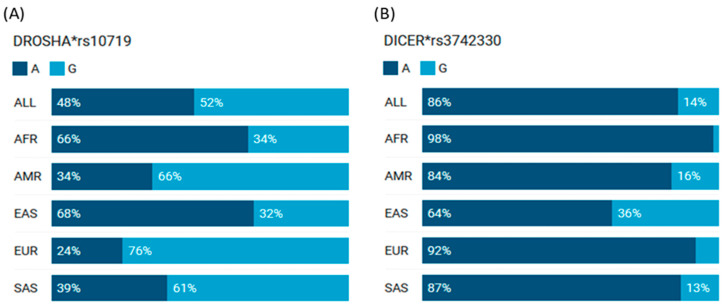
The allele frequencies of *DROSHA* rs10719 and *DICER* rs3742330 variants in different populations. (**A**) *DROSHA* (**B**) *DICER*. AMR: American; AFR: African; EAS: East Asian; EUR: European; SAS: South Asians. Data source: 1000 Genomes Project Phase 3 allele frequencies [Ensembl.org] (last accessed on 20 March 2024)”. *rs: reference sequence.

Genetic variants in *DROSHA* and *DICER* genes at 5p13.3 and 14q32.13 were screened. The top cited single-nucleotide variants were selected, and the variant with the highest citation and minor allele frequency over 0.1 was analyzed in the current study (Supplementary Table S1). Allelic frequencies of *DROSHA* rs10719 and *DICER* rs3742330 across different ethnic populations are shown in Figure 1.

**Figure 2:**
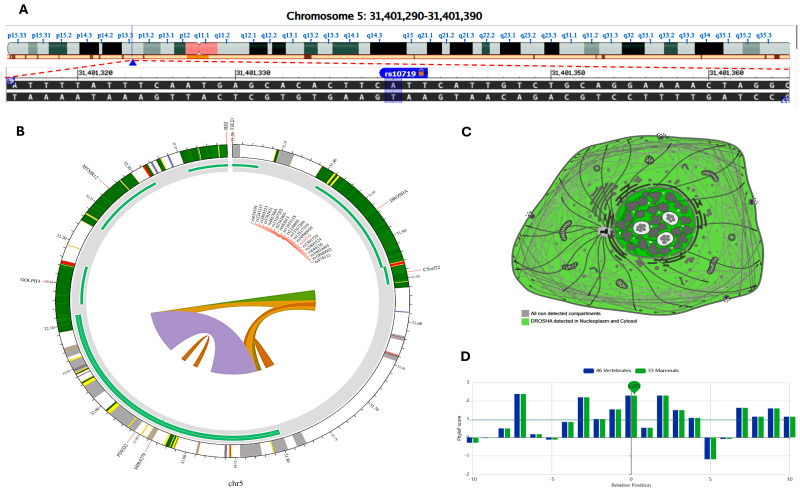
Analysis of the *DROSHA* gene structure and its associated 3D interactions with other genes and variants mediated by chromatin loops. (**A**) The *DROSHA* gene is on the short arm of chromosome 5 on the forward strand, following the ‘GRCh38.p14’ assembly. The rs10719 variant is positioned at 5:31,401,340 (highlighted), where the ancestral nucleotide ‘A’ is replaced by the alternative (minor) allele ‘G’ (https://www.ncbi.nlm.nih.gov/snp/rs10719). (**B**) A Circos plot illustrating the chromatin loops and other 2D characteristics related to the variant of interest, generated using 3DSNP 2.0 (https://omic.tech/3dsnpv2/). The plot displays, from the outer edge to the inner section, the chromatin states, annotated genes, the current SNP of interest and associated SNPs, and 3D chromatin interactions. A color key corresponding to the chromatin states and loops for twelve distinct cell types has been detailed previously [23]. (**C**) *DROSHA*’s subcellular localization can be accessed via https://www.proteinatlas.org/ENSG00000113360-DROSHA/subcellular. (**D**) The conservation score for the variant of interest is recorded as 2.277, derived from multiple alignments of vertebrate (*n* = 46) and mammalian (*n* = 33) genomes. All databases were last accessed on 30 March 2024.

The *DROSHA* ribonuclease III gene (ENSG00000113360) is located on chromosome 5p13.3: 31,400,494–31,532,093 (reverse strand, Figure 2A). It is implicated in the initial processing step of microRNA (miRNA) biogenesis (gene ontology (GO): 0006396). The studied variant rs10719 (NC_000005.10: g.31401340 A: G) is present in the three prime untranslated region. A customizable ‘Circos plotting system’ was established utilizing “3DSNP 2.0′ (https://omic.tech/3dsnpv2/)” (accessed 30 March 2024) to visualize the 3D chromatin structure along with a selection of crucial chromatin marks around the SNP of interest (Figure 2B). For predicting transcription factors, breast tissue was chosen as the cell type, while epithelial cells were selected for the assessment of histone modification. The primary subcellular localization of *DROSHA*, with a high confidence level, is found in the nucleoplasm and cytosol (Figure 2C). Its “PhyloP conservation score” is 2.277 (Figure 2D). The absolute values of this score correspond to “−log(*p*-value) under a null hypothesis of neutral evolution”. Positive scores indicate predicted conserved sites, whereas negative scores are assigned to regions anticipated to evolve rapidly.

**Figure 3:**
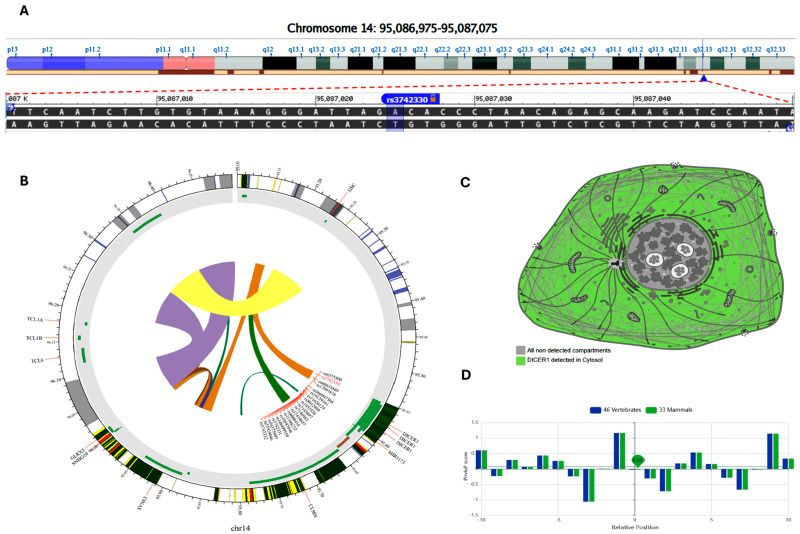
Structural analysis of the *DICER* gene and its associated 3D interactions with other genes and variants mediated by chromatin loops. (**A**) The DICER gene is situated on the long arm of chromosome 14 on the reverse strand, aligning with the ‘GRCh38.p14’ assembly. The rs3742330 variant is found at position 14: 95,087,025 (highlighted), where the ancestral nucleotide ‘A’ is replaced by the alternative (minor) allele ‘G’ (https://www.ncbi.nlm.nih.gov/snp/rs3742330). (**B**) A Circos plot displaying the chromatin loops and other 2D features related to the variant of interest was created using “3DSNP 2.0 (https://omic.tech/3dsnpv2/)”. The plot illustrates, from the outermost section to the inner, the chromatin states, annotated genes, the currently examined SNP and its associated SNPs, and 3D chromatin interactions. The color key for chromatin states and loops across twelve different cell types is provided in a previous work [23]. (**C**) Information regarding the subcellular distribution of *DICER* can be accessed at https://www.proteinatlas.org/ENSG00000100697-DICER1/subcellular. (**D**) The conservation score for the variant of interest is reported as −0.023, derived from multiple alignments of vertebrate (*n* = 46) and mammalian (*n* = 33) genomes. All databases were last accessed on 30 March 2024.

Regarding the *DICER* ribonuclease III gene (ENSG00000100697), it is located on chromosome 14q32.13: 95,086,228–95,158,010 (reverse strand, Figure 3A). It is implicated in short dsRNA-mediated post-transcriptional gene silencing (GO:0006396). The studied variant rs3742330 (NC_000014.9:95087024 A: G) is present in the three prime untranslated region. A customizable Circos plotting system was created utilizing “3DSNP 2.0 (https://omic.tech/3dsnpv2/)” (accessed 30 March 2023) to visualize the 3D chromatin topology, along with crucial chromatin marks surrounding the SNP of interest (Figure 3B), based on the previously mentioned selection criteria. The primary subcellular localization of *DICER*, with a high degree of confidence, is found in the cytosol (Figure 3C). Its “PhyloP conservation score” is −0.023 (Figure 3D).

### 3.2. Pooled Analysis for the Role of DROSHA rs10719 and DICER rs3742330 SNPs in Cancer

We found 10 and 16 original articles on *DROSHA* rs10719 and *DICER* rs3742330 and different types of cancers (Supplementary Table S2). Pairwise comparison for the G allele versus the A allele showed rs10719 G to be associated with a lower risk of chronic lymphocytic leukemia (OR = 0.65, 95%CI = 0.44–0.97, *p =* 0.038). However, rs3742330 G was associated with contradictory results, showing higher risk in laryngeal cancer (OR = 1.41, 95%CI = 1.01–1.98, *p* = 0.047) and conferred protection against gastric cancer (OR = 0.76, 95%CI = 0.64–0.91, *p* = 0.002) and cervical precancerous lesions (OR = 0.73, 95%CI = 0.57–0.92, *p =* 0.009) (Supplementary Table S3).

### 3.3. Characteristics of the Tissue Samples

This study included two different cohorts. For the first cohorts, 103 paired FFPE tissue samples were compared. Patient characteristics are shown in Table 1.

### 3.4. Genotype/Allele Frequencies in Tissue Samples

Allelic discrimination analysis of 103 paired tissue samples of women with BC revealed minor allele frequencies of 0.39 for *DROSHA* G and 0.46 for *DICER* A. Genotype frequencies followed the Hardy–Weinberg equilibrium (*p* > 0.05). Screening the overall frequencies of *DROSHA* rs10719 and *DICER* rs3742330 genotypes showed the association of both variants with disease susceptibility. Carrying rs10719 G or rs3742330 A conferred protection against the development of BC under heterozygote comparison, homozygote comparison, and allelic models (Table 2).

**Figure 4:**
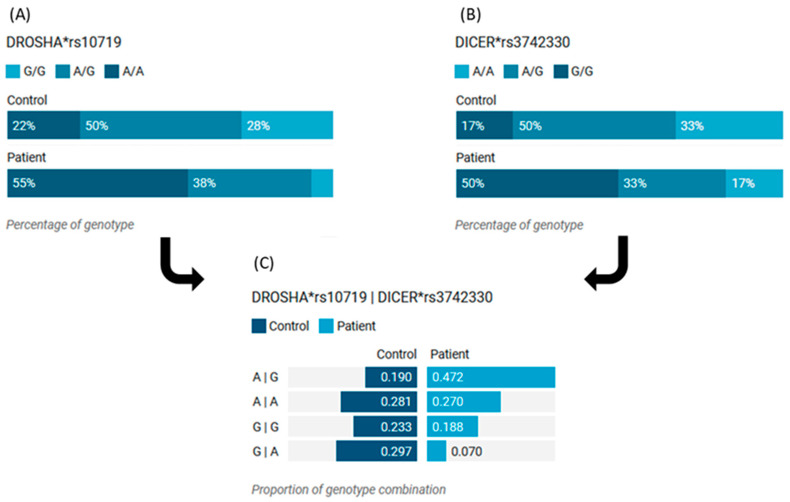
Genotype combination analysis of *DICER* and *DROSHA* genes in tissues of BC women. (**A**) Distribution of *DROSHA* genotypes in patients and controls. (**B**) Distribution of *DICER* genotypes in patients and controls. (**C**) Distribution of combined *DROSHA* and *DICER* genotypes in patients and controls. *rs: reference sequence.

Similarly, patients with both *DROSHA* A and *DICER* G genotype combinations were associated with the risk of BC (Figure 4).

### 3.5. Somatic Mutation Rate in Paired Tissue Samples

**Figure 5:**
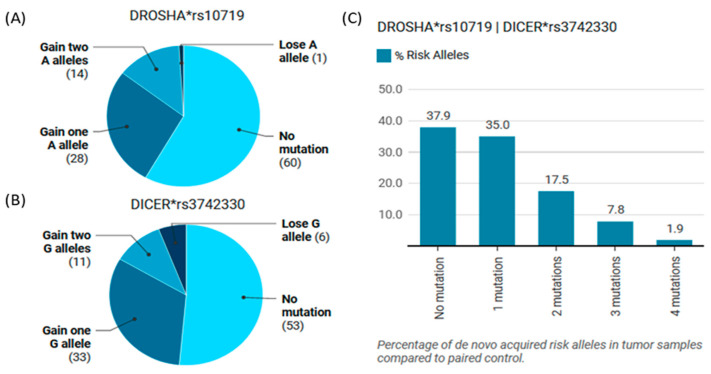
Somatic mutation analyses of *DICER* and *DROSHA* polymorphisms in paired tissues of women with BC. (**A**) Genotype alteration of the *DROSHA* gene in cancer and non-cancer tissues. The A allele is considered the risky variant. (**B**) Genotype alteration of *DICER* gene in cancer and non-cancer tissues. G allele is considered the risky variant. (**C**) Genotype alteration of combined *DROSHA* and *DICER* genes in cancer/non-cancer tissues. *rs: reference sequence.

Paired analysis of tumor/non-tumor tissues per each patient displayed significant differences between the two types of tissue samples (*p* <0.001). The rates of conversion and somatic mutation load are depicted in Figure 5 For the *DROSHA* gene, 28 tumor samples (27.2%) acquired a single A allele, while 14 samples (13.6%) showed the conversion of the two alleles from G/G to A/A with a total rate of conversion from G to A of 40.8%. In contrast, 60 patients (58.3) have similar genotypes to the adjacent non-cancer tissues (Figure 5A). Regarding the *DICER* gene, they exhibited a similar conversion rate (42.7%); indeed, 33 (32%) and 11 (10.7%) tumor samples showed one and two hit mutations from A to G, respectively, while 53 patients (51.5%) had the same genotype of neighboring tissues (Figure 5B). One and six samples represented a reverse direction of allelic conversion in *DROSHA* and *DICER* genes. The seven cohorts with the reverse direction exhibited the acquisition of risk alleles in the alternative gene. The de novo acquisition of risky alleles in both genes accounted for 64 women (37.9%) (Figure 5C).

### 3.6. Assessment of the Prognostic Value of DROSHA and DICER Genotyping in BC Tissues

Univariate analysis showed no significant association of *DROSHA* and *DICER* gene polymorphisms with clinical and pathological outcomes (Table 3 and Table 4).

Similarly, no significant difference existed between tumors with and without conversion to risky alleles and their paired adjacent non-cancer samples (Table 5).

### 3.7. Survival Analysis

Median disease-free survival was 15 months (IQR = 8.0–20.0), and the overall survival time was 17 months (IQR = 14.0–20.0). Comparison between cohorts with and without conversion of risky alleles showed insignificant results. For *DROSHA*, the median disease-free survival/overall survival times were 15.2 months (95%CI = 12.9–17.4) in the mutant group versus 15.6 (95%CI = 13.7–17.4) months in non-mutant group (*p* = 0.76) and 19.3 months (95%CI = 18.4–20.1) in the mutant group versus 19.4 (95%CI = 18.6–20.1) months in non-mutant group (*p* = 0.75), respectively. Regarding *DICER* SNP, the median disease-free survival and overall survival times were 15.8 months (95%CI = 13.5–17.9) in the mutant group versus 15.2 (95%CI = 13.3–17.1) months in non-mutant group (*p* = 0.76) and 19.3 months (95%CI = 18.6–20.1) in the mutant group versus 19.3 (95%CI = 18.4–20.1) months in non-mutant group (*p* = 0.68), respectively.

### 3.8. Characteristics of the Study Population with Blood Samples

For the second cohort, the blood samples of patients and controls were compared. The characteristics of the study populations are depicted in Table 6. Blood analysis was performed to pinpoint their role in the cancer screening of inherited risk alleles.

**Figure 6:**
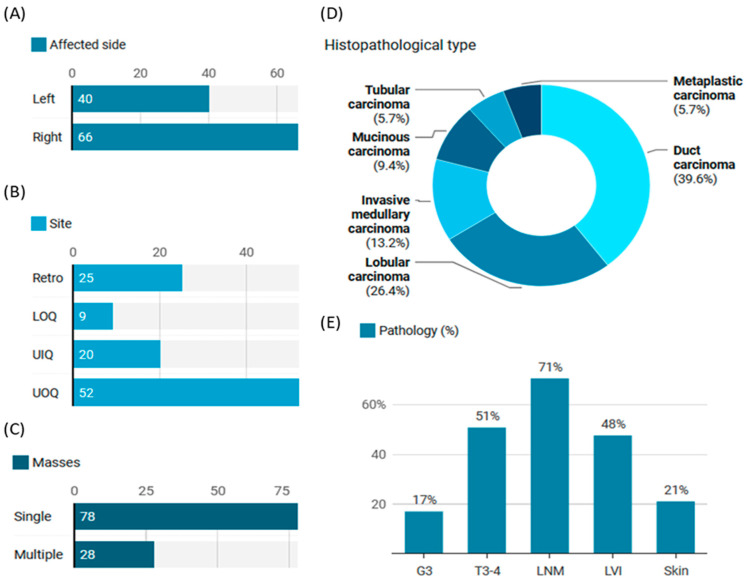
Clinical presentation of BC women with blood samples (*n* = 106). (**A**) Counts of affected sides. (**B**) Counts of affected locations. (**C**) Counts of patients according to the number of masses at the time of presentation. (**D**) The different histopathological types. (**E**)Pathology-related data of patients with BC and provided blood samples. G3: Grade 3, T3-4: stage 3-4, LNM: Lymph node metastasis, LVI: lymphovascular infiltration.

The most common presenting symptoms at the time of presentation were breast lump (41.5%) and mastalgia (14.6%), followed by nipple (8.5%) and skin changes (7.5%). Most lesions were presented on the right side (*n* = 66, 62.3%) and in the upper outer quadrant (*n* = 52, 49.1%). Of the affected women, 28 (26.4%) presented with multiple masses, and 71% had positive lymph node infiltration (Figure 6).

### 3.9. Genotype and Allele Frequencies in Blood Samples

**Figure 7:**
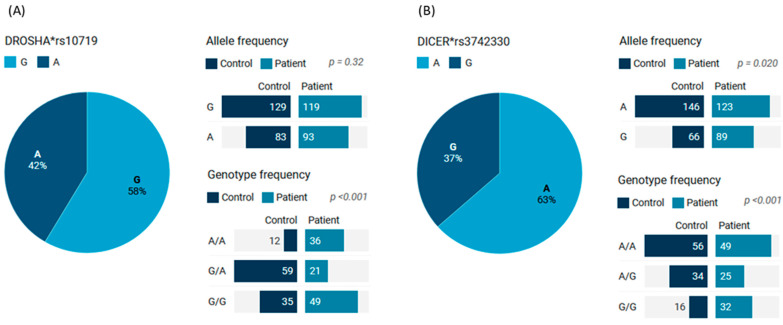
The genotype and allele frequencies of the *DICER* and *DROSHA* genes in blood samples of BC and non-cancer women: (**A**) *DROSHA* rs10719; (**B**) *DICER* rs3742330. Pie charts represented the percentage of each allele in the overall cohorts (cases and controls). The bar chart showed the frequencies (counts) of cohorts per allele or genotype. A two-sided Chi-square test was used. Significance was set at *p* < 0.05. *rs: reference sequence.

Minor allele frequencies were 0.42 for the *DROSHA* A and 0.37 for the *DICER* G alleles. As shown in Figure 7, the most common genotype in patients was DROSHA*G/G, accounting for 46.2% (*n* = 49), whereas in controls, the heterozygote form A/G was the most prevalent (*n* = 59, 55.7%); *p* < 0.001. As for the *DICER* genotype, A/A was the most frequent in patients (*n* = 49, 46.2%) and controls (*n* = 56, 52.8%). However, G/G carriers were twice as common as in controls (30.2% vs. 15.1%, *p* < 0.001).

### 3.10. Association of DICER and DROSHA Polymorphisms in Blood with BC Risk

**Figure 8:**
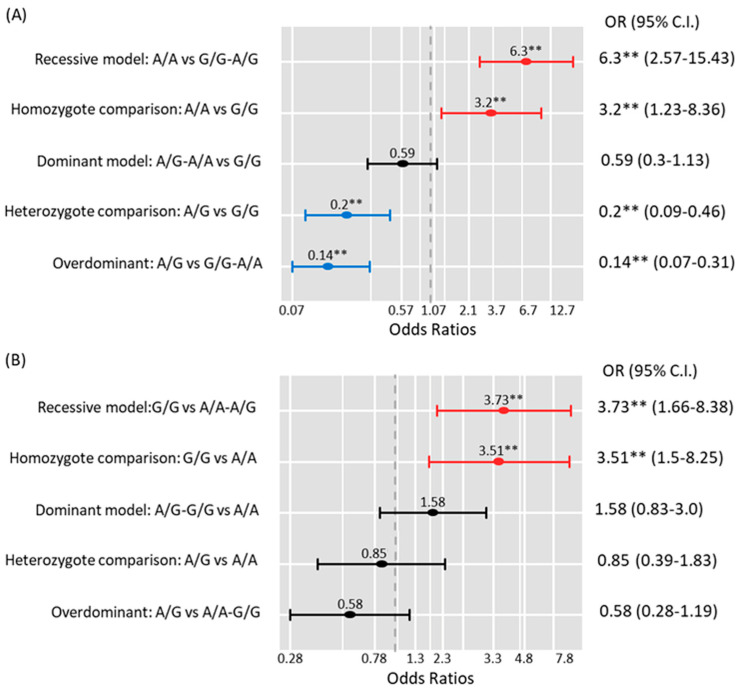
Genetic association models for disease risk assessment: (**A**) *DROSHA* rs10719; (**B**) *DICER* rs3742330. Multivariate regression models were performed and shown as odds ratios (ORs) and 95% confidence intervals (95%CI). Adjusted variables were patient age at diagnosis, marital status, occupation, a family history of cancer, prior breast problems, smoking, body mass index, diabetes, hypertension, and hepatitis C virus infection. The red line is a risky genotype, the blue line is a protective genotype, and the black line is insignificant. ** indicate significance at *p*-value < 0.05.

Genetic association model analysis showed that cohorts who were carrying *DROSHA* A/A were at higher risk of developing BC under the recessive model (OR = 6.3, 95%CI = 1.23–8.36, *p* < 0.001) and homozygote comparison model (OR = 3.2, 95%CI = 1.23–9.36, *p* < 0.001). In contrast, carrying the heterozygote form (A/G) ameliorates the impact of the risk allele under the heterozygote comparison model (OR = 0.20, 95%CI = 0.09–0.46, *p* < 0.001) and over-dominant model (OR = 0.14, 95%CI = 0.07–0.31, *p* < 0.001) (Figure 8A). For *DICER* gene risk assessment, G/G cohorts had over three times more risk of disease susceptibility under the recessive model (OR = 3.73,95%CI = 1.66–8.38, *p* = 0.001) and homozygote comparison model (OR = 3.51, 95%CI = 1.5–8.25) (Figure 8B). Carriers of both risk alleles (*DROSHA* A and *DICER* G) conferred higher susceptibility for developing BC (OR = 2.18, 95%CI = 1.23–3.89); *p* = 0.008 (Table 7).

### 3.11. DICER and DROSHA Polymorphisms as a Prognostic Marker

For the BC cohorts with blood samples, we did not find a significant association with the *DROSHA* and *DICER* genotypes or risk alleles with clinical and pathological features in cancer-affected women using univariate and multivariate analyses. Adjusted variables for logistic regression models were age, a family history of cancer, prior breast problems, smoking, body mass index, diabetes, hypertension, and hepatitis C virus infection (Table 8).

## 4. Discussion

In this study, we investigated the association of two common polymorphisms in the miRNA processing genes *DROSHA* (rs10719) and *DICER* (rs3742330) with BC risk and clinical outcomes in an Egyptian population. We found that both SNPs were significantly associated with altered BC susceptibility, with the *DROSHA* rs10719 A allele and the *DICER* rs3742330 G allele conferring increased risk. Furthermore, a higher frequency of the risk alleles was detected in breast tumor tissues compared to adjacent normal tissues, suggesting a potential role for these variants in driving miRNA dysregulation during breast tumorigenesis. However, neither SNP showed significant associations with clinicopathological characteristics or survival outcomes.

Initially, in our observations regarding the lateralization of BC, there was a predominance of cases (approximately two-thirds of cases) affecting the right side, although they showed insignificant association with the clinicopathological characteristics of the study population (Table 3, Table 4 and Table 5); this may provide additional insights into the biological factors influencing tumorigenesis. This lateralization could reflect anatomical, hormonal, or environmental factors, which may warrant further investigation [24]. Understanding whether the affected side correlates with specific genetic makeup or tumor biology may enhance our comprehension of BC heterogeneity. Although the predominance of right-sided tumors does not alter our primary findings regarding the associations of the studied variants with BC risk, it suggests an avenue for future research to explore the implications of tumor location concerning genetic predispositions [25].

*DROSHA* and *DICER* are essential endoribonucleases involved in the biogenesis of microRNAs (miRNAs), which play pivotal roles in regulating gene expression and various cellular processes [26]. *DROSHA* processes primary miRNA transcripts (pri-miRNAs) into precursor miRNAs (pre-miRNAs) in the nucleus. The functioning of *DROSHA* is vital as it ensures that the remaining pre-miRNA is appropriately sized for subsequent processing by *DICER*. Any alterations in *DROSHA* activity, such as those potentially conferred by the rs10719 variant, might disrupt this initial step of miRNA maturation, leading to aberrant levels of downstream miRNAs [27]. *DICER*, on the other hand, processes pre-miRNAs into mature miRNAs in the cytoplasm and is also involved in the generation of small interfering RNAs (siRNAs) [28]. The *DICER* rs3742330 variant may influence *DICER*’s enzymatic efficiency, affecting the quantity and quality of generated mature miRNAs. This disruption can have significant downstream consequences, including the altered expression of target mRNAs involved in critical cellular pathways such as apoptosis, proliferation, and differentiation [29]. For instance, the dysregulation of miRNAs processed through these pathways can lead to the misregulation of oncogenes and tumor suppressor genes, thereby promoting tumorigenesis. Specific miRNAs that *DROSHA* and *DICER* can potentially impact include oncogenic miRNAs like miR-21 and tumor suppressor miRNAs such as let-7. Elevated levels of oncogenic miRNAs can facilitate cancer cell proliferation and survival by inhibiting pro-apoptotic signals. Conversely, a deficit in tumor suppressor miRNAs can lead to the inhibition of oncogenes, further driving cancer progression [30]. Thus, understanding the genetic variants in miRNA processing genes like *DROSHA* and *DICER* not only provides insights into BC susceptibility but also highlights the importance of downstream miRNA-mediated regulatory networks in the development and progression of BC.

Our findings on the association of *DROSHA* rs10719 with increased BC risk align with a previous study by Jiang et al., which reported a higher frequency of the AA genotype in patients with BC compared to healthy controls in a Chinese population [16]. Also, it has been associated with colorectal and bladder cancers in previous reports [31,32]. Similarly, the association of the *DICER* rs3742330 G allele with cancer risk has been observed in several other malignancies, including colorectal [31,33], gastric [34,35], hepatocellular [36], prostate [37], larynx [38], and thyroid [39] cancers, as well as precancerous cervical lesion [40].

The functional consequences of these SNPs for miRNA processing efficiency and target gene regulation remain to be fully elucidated. However, it has been proposed that the rs10719 variant may affect *DROSHA*’s mRNA stability and alter its subcellular localization [41]. Interestingly, this SNP is located in the miR-27b binding site within *DROSHA* 3′UTR [42], which has been proven to be oncogenic in MCF7 BC cells and may have tumor suppressive activity under certain conditions, as evidenced by a “CRISPR/Cas9 deletion study” conducted by Hannafon et al. [43]. At the same time, the rs3742330 variant may influence *DICER* mRNA expression and enzymatic activity [42]. As this SNP is located within the potential target sequences of miR-632, miR-3622a-5p, and miR-5582-5p, it may affect cellular processes like apoptosis, cell growth, and migration/invasion [44]. Mir-632 was found to be a putative epigenetic down-regulator of DNAJB6, a constitutive member of the heat shock protein 40 family, which supports BC oncogenesis and progression [45]. MiR-3622b-5p has been reported to impact the Her2-positive BC cell line negatively, and miR-5582-5p, via the long noncoding RNA LUCAT1/miR-5582-3p/TCF7L2 axis, was associated with the regulatory mechanisms of BC stemness [46]. The *DICER* rs3742330 G variant enhances the affinity of these microRNAs for the DICER 3′ UTR, reducing DICER expression. This decrease results in diminished RNA cleavage and translation repression, along with heightened cell migration, invasion, and angiogenesis, all of which may impact the progression of BC [47]. This could support the present study findings that carrying the G allele and the GG genotype confer higher susceptibility to developing BC in our cohort.

The higher frequency of risk alleles in tumor tissues than matched normal tissues suggests that the studied variants may undergo positive selection during BC development, potentially conferring a growth advantage to tumor cells. This finding is consistent with the concept of “onco-miRNAs”, whereby dysregulated miRNA networks can promote various hallmarks of cancer, such as sustained proliferation, the evasion of apoptosis, and metastatic dissemination [48]. However, it is essential to note that the observed differences in genotype frequencies between tumor and normal tissues could also be influenced by factors such as tumor purity, genetic heterogeneity, and tissue-specific mosaicism [49,50,51,52].

The lack of significant associations between the studied SNPs and clinicopathological features or survival outcomes in our cohort suggests that these variants may primarily influence BC initiation rather than progression [52,53,54]. However, it is also possible that the impacts of these SNPs on clinical outcomes may be modulated by other genetic, epigenetic, or environmental factors not accounted for in our analysis [55,56]. Additionally, the relatively small sample size and short follow-up duration of our study may have limited our ability to detect subtle associations with survival endpoints.

Our study has several strengths, including the comprehensive evaluation of both blood and tissue samples, including age-matched healthy controls, and the detailed clinicopathological annotation of BC cases. However, we acknowledge certain limitations that should be considered when interpreting our results. First, our study was conducted in a single institution and may not fully represent the Egyptian population. Second, we did not have information on potential confounding factors such as lifestyle habits and environmental exposures, which could have influenced the observed associations. Third, we did not perform functional experiments to validate the biological consequences of the studied SNPs on miRNA processing efficiency or target gene expression.

## 5. Conclusions

Our study presents evidence linking the *DROSHA* rs10719 and *DICER* rs3742330 polymorphisms to an elevated risk of breast cancer in an Egyptian cohort, suggesting that these variants may play a role in miRNA dysregulation during breast tumorigenesis. These findings underscore the significance of exploring genetic variability within miRNA processing pathways and its influence on cancer susceptibility and progression. To validate our results and clarify the associated molecular mechanisms, further large-scale research across diverse populations, along with functional analyses of the identified variants, is essential. Ultimately, enhancing our understanding of the genetic factors contributing to miRNA dysregulation in breast cancer could facilitate the development of novel risk assessment tools, prognostic biomarkers, and targeted therapeutic interventions.

## Figures and Tables

**Table 1:** Patients with FFPE tissue sample demographics and clinical features.

Characteristics	Levels	Tissue Cohorts (*n* = 103)
Age	<45 years	51 (49.5)
	≥45 years	52 (50.5)
Affected side	Left	38 (36.9)
	Right	65 (63.1)
Site	Retro	24 (23.3)
	LOQ	10 (9.7)
	UIQ	23 (22.3)
	UOQ	46 (44.7)
Number of masses	Single	84 (81.6)
	Multiple	19 (18.4)
Histopathological type	Duct carcinoma	39 (37.9)
	Lobular carcinoma	23 (22.3)
	Invasive medullary carcinoma	17 (16.5)
	Mucinous carcinoma	13 (12.6)
	Tubular carcinoma	6 (5.8)
	Metaplastic carcinoma	5 (4.9)
Grade	G2	82 (79.6)
	G3	21 (20.4)
T stage	T2	53 (51.5)
	T3	22 (21.4)
	T4	28 (27.2)
N stage	N0	22 (21.4)
	N1	38 (36.9)
	N2	37 (35.9)
	N3	6 (5.8)
M stage	M0	47 (45.6)
	M1	43 (41.7)
	Mx	13 (12.6)
LVI	Negative	54 (52.4)
	Positive	49 (47.6)
Skin infiltration	Negative	85 (82.5)
	Positive	18 (17.5)
Clinical stage	IIA	16 (15.5)
	IIB	15 (14.6)
	IIIA	13 (12.6)
	IIIB	16 (15.5)
	IV	43 (41.7)
NPI	Good	48 (46.6)
	Poor	55 (53.4)
ESMO	Low risk	36 (35)
	High risk	67 (65)
ER/PR	Negative	39 (37.9)
	Positive	64 (62.1)
HER2+	Negative	81 (78.6)
	Positive	22 (21.4)
Molecular subtype	Luminal A	50 (48.5)
	Luminal B	14 (13.6)
	HER2+	8 (7.8)
	TNBC	31 (30.1)
IHP	Good	64 (62.1)
	Moderate	31 (30.1)
	Poor	8 (7.8)
Recurrence	Negative	48 (46.6)
	Positive	55 (53.4)
Survival	Alive	42 (40.8)
	Dead	61 (59.2)

Data are presented as counts (percentages). LVI: lymphovascular infiltration; ER/PR: estrogen and progesterone receptor; HER2+: HER2/neu receptor; NPI: Nottingham Prognostic Index, calculated as [0.2 × tumor size in cm] + tumor grade [1,2,3] + lymph node stage [1–3, according to stages A–C]; ESMO: European Society of Medical Oncology; IHPI: Immunohistochemical Prognostic Index estimated based on the three-receptor status (HER2, ER, and PR). A two-sided Chi-square test was used. Significance was set at *p* < 0.05.

**Table 2:** Genotype and allele frequencies of *DICER* and *DROSHA* genes in tissues of BC women.

SNP ID	Variant	Controls (*n* = 103)	Patients (*n* = 103)	*p*-Value	Adjusted OR (95%CI)
*DROSHA* rs10719	Genotypes				
	A/A	23 (22.3%)	57 (55.3%)	**<0.001**	Reference
	A/G	51 (49.5%)	39 (37.9%)		0.31 (0.16–0.58)
	G/G	29 (28.2%)	7 (6.8%)		0.10 (0.04–0.25)
	Alleles				
	A	97 (48)	153 (74)	**<0.001**	Reference
	G	107 (52)	53 (26)		0.32 (0.21–0.48)
*DICER* rs3742330	Genotypes				
	G/G	18 (17.5%)	51 (49.5%)	**<0.001**	Reference
	A/G	51 (49.5%)	34 (33%)		0.23 (0.11–0.46)
	A/A	34 (33%)	18 (17.5%)		0.18 (0.08–0.40)
	Alleles				
	G	87 (42)	136 (66)	**<0.001**	Reference
	A	119 (58)	70 (34)		0.37 (0.25–0.56)
*DROSHA* (A/G)|*DICER* (A/G)	Allele combination				
	A|G	0.1899	0.4724	**<0.001**	Reference
	A|A	0.281	0.2703		0.43 (0.23–0.81)
	G|G	0.2325	0.1878		0.34 (0.17–0.72)
	G|A	0.2966	0.0695		0.10 (0.05–0.21)

Data are presented as counts (percentages) or proportions. A two-sided Chi-square test was used. The regression model was adjusted by patient age at diagnosis. Heterozygote comparison, homozygote comparison, and allelic models are shown as odds ratios (ORs) and 95% confidence intervals (95%CIs). Bold values indicate significance at *p* < 0.05.

**Table 3:** Association of the *DROSHA* variant with clinical and pathological features.

Characteristics	Levels	A/A Genotype (*n* = 57)	A/G Genotype (*n* = 39)	G/G Genotype (*n* = 7)	*p*-Value
Age	<45 years	30 (52.6)	19 (48.7)	2 (28.6)	0.48
	≥45 years	27 (47.4)	20 (51.3)	5 (71.4)	
Affected side	Left	9 (31)	20 (39.2)	9 (39.1)	0.74
	Right	20 (69)	31 (60.8)	14 (60.9)	
Site	Retro	12 (21.1)	11 (28.2)	1 (14.3)	0.90
	LOQ	5 (8.8)	4 (10.3)	1 (14.3)	
	UIQ	15 (26.3)	7 (17.9)	1 (14.3)	
	UOQ	25 (43.9)	17 (43.6)	4 (57.1)	
Number of masses	Single	46 (80.7)	31 (79.5)	7 (100)	0.42
	Multiple	11 (19.3)	8 (20.5)	0 (0)	
Histopathological type	Duct carcinoma	22 (38.6)	15 (38.5)	2 (28.6)	0.91
	Lobular carcinoma	13 (22.8)	8 (20.5)	2 (28.6)	
	Invasive medullary carcinoma	9 (15.8)	6 (15.4)	2 (28.6)	
	Mucinous carcinoma	6 (10.5)	6 (15.4)	1 (14.3)	
	Tubular carcinoma	5 (8.8)	1 (2.6)	0 (0)	
	Metaplastic carcinoma	2 (3.5)	3 (7.7)	0 (0)	
Grade	G2	49 (86)	28 (71.8)	5 (71.4)	0.21
	G3	8 (14)	11 (28.2)	2 (28.6)	
T stage	T2	34 (59.6)	15 (38.5)	4 (57.1)	0.35
	T3	10 (17.5)	11 (28.2)	1 (14.3)	
	T4	13 (22.8)	13 (33.3)	2 (28.6)	
N stage	N0	13 (22.8)	8 (20.5)	1 (14.3)	0.71
	N1	19 (33.3)	17 (43.6)	2 (28.6)	
	N2	23 (40.4)	11 (28.2)	3 (42.9)	
	N3	2 (3.5)	3 (7.7)	1 (14.3)	
M stage	M0	27 (47.4)	19 (48.7)	1 (14.3)	0.19
	M1	21 (36.8)	18 (46.2)	4 (57.1)	
	Mx	9 (15.8)	2 (5.1)	2 (28.6)	
LVI	Negative	29 (50.9)	22 (56.4)	3 (42.9)	0.75
	Positive	28 (49.1)	17 (43.6)	4 (57.1)	
Skin infiltration	Negative	48 (84.2)	31 (79.5)	6 (85.7)	0.81
	Positive	9 (15.8)	8 (20.5)	1 (14.3)	
Clinical stage	IIA	10 (17.5)	5 (12.8)	1 (14.3)	0.87
	IIB	9 (15.8)	6 (15.4)	0 (0)	
	IIIA	9 (15.8)	3 (7.7)	1 (14.3)	
	IIIB	8 (14)	7 (17.9)	1 (14.3)	
	IV	21 (36.8)	18 (46.2)	4 (57.1)	
NPI	Good	27 (47.4)	19 (48.7)	2 (28.6)	0.61
	Poor	30 (52.6)	20 (51.3)	5 (71.4)	
ESMO	Low risk	20 (35.1)	14 (35.9)	2 (28.6)	0.93
	High risk	37 (64.9)	25 (64.1)	5 (71.4)	
ER/PR	Negative	23 (40.4)	13 (33.3)	3 (42.9)	0.75
	Positive	34 (59.6)	26 (66.7)	4 (57.1)	
HER2+	Negative	45 (78.9)	31 (79.5)	5 (71.4)	0.89
	Positive	12 (21.1)	8 (20.5)	2 (28.6)	
Molecular subtype	Luminal A	27 (47.4)	20 (51.3)	3 (42.9)	0.97
	Luminal B	7 (12.3)	6 (15.4)	1 (14.3)	
	HER2+	5 (8.8)	2 (5.1)	1 (14.3)	
	TNBC	18 (31.6)	11 (28.2)	2 (28.6)	
IHP	Good	34 (59.6)	26 (66.7)	4 (57.1)	0.89
	Moderate	18 (31.6)	11 (28.2)	2 (28.6)	
	Poor	5 (8.8)	2 (5.1)	1 (14.3)	
Recurrence	Negative	28 (49.1)	17 (43.6)	3 (42.9)	0.85
	Positive	29 (50.9)	22 (56.4)	4 (57.1)	
Survival	Alive	25 (43.9)	15 (38.5)	2 (28.6)	0.69
	Dead	32 (56.1)	24 (61.5)	5 (71.4)	

Data are presented as counts (percentage). LVI: lymphovascular infiltration; ER/PR: estrogen and progesterone receptors; HER2+: HER2/neu receptor; NPI: Nottingham Prognostic Index, calculated as [0.2 × tumor size in cm] + tumor grade [1,2,3] + lymph node stage [1–3, according to stages A–C]; ESMO: European Society of Medical Oncology; IHPI: Immunohistochemical Prognostic Index estimated based on the three-receptor status (HER2, ER, and PR). A two-sided Chi-square test was used. Significance was set at *p* < 0.05.

**Table 4:** Association of the *DICER* polymorphism with clinical and pathological features.

Characteristics	Levels	A/A Genotype (*n* = 18)	A/G Genotype (*n* = 34)	G/G Genotype (*n* = 51)	*p*-Value
Age	<45 years	10 (55.6)	18 (52.9)	23 (45.1)	0.66
	≥45 years	8 (44.4)	16 (47.1)	28 (54.9)	
Affected side	Left	11 (32.4)	20 (39.2)	7 (38.9)	0.79
	Right	23 (67.6)	31 (60.8)	11 (61.1)	
Site	Retro	2 (11.1)	9 (26.5)	13 (25.5)	0.79
	LOQ	2 (11.1)	2 (5.9)	6 (11.8)	
	UIQ	4 (22.2)	7 (20.6)	12 (23.5)	
	UOQ	10 (55.6)	16 (47.1)	20 (39.2)	
Number of masses	Single	15 (83.3)	26 (76.5)	43 (84.3)	0.64
	Multiple	3 (16.7)	8 (23.5)	8 (15.7)	
Histopathological type	Duct carcinoma	8 (44.4)	11 (32.4)	20 (39.2)	0.95
	Lobular carcinoma	4 (22.2)	9 (26.5)	10 (19.6)	
	Invasive medullary carcinoma	2 (11.1)	5 (14.7)	10 (19.6)	
	Mucinous carcinoma	2 (11.1)	5 (14.7)	6 (11.8)	
	Tubular carcinoma	2 (11.1)	2 (5.9)	2 (3.9)	
	Metaplastic carcinoma	0 (0)	2 (5.9)	3 (5.9)	
Grade	G2	16 (88.9)	24 (70.6)	42 (82.4)	0.24
	G3	2 (11.1)	10 (29.4)	9 (17.6)	
T stage	T2	9 (50)	15 (44.1)	29 (56.9)	0.29
	T3	2 (11.1)	11 (32.4)	9 (17.6)	
	T4	7 (38.9)	8 (23.5)	13 (25.5)	
N stage	N0	5 (27.8)	6 (17.6)	11 (21.6)	0.52
	N1	7 (38.9)	10 (29.4)	21 (41.2)	
	N2	6 (33.3)	14 (41.2)	17 (33.3)	
	N3	0 (0)	4 (11.8)	2 (3.9)	
M stage	M0	10 (55.6)	14 (41.2)	23 (45.1)	0.75
	M1	5 (27.8)	16 (47.1)	22 (43.1)	
	Mx	3 (16.7)	4 (11.8)	6 (11.8)	
LVI	Negative	11 (61.1)	14 (41.2)	29 (56.9)	0.26
	Positive	7 (38.9)	20 (58.8)	22 (43.1)	
Skin infiltration	Negative	15 (83.3)	27 (79.4)	43 (84.3)	0.84
	Positive	3 (16.7)	7 (20.6)	8 (15.7)	
Clinical stage	IIA	4 (22.2)	4 (11.8)	8 (15.7)	0.43
	IIB	1 (5.6)	4 (11.8)	10 (19.6)	
	IIIA	3 (16.7)	6 (17.6)	4 (7.8)	
	IIIB	5 (27.8)	4 (11.8)	7 (13.7)	
	IV	5 (27.8)	16 (47.1)	22 (43.1)	
NPI	Good	10 (55.6)	12 (35.3)	26 (51)	0.26
	Poor	8 (44.4)	22 (64.7)	25 (49)	
ESMO	Low risk	6 (33.3)	10 (29.4)	20 (39.2)	0.64
	High risk	12 (66.7)	24 (70.6)	31 (60.8)	
ER/PR	Negative	6 (33.3)	17 (50)	16 (31.4)	0.20
	Positive	12 (66.7)	17 (50)	35 (68.6)	
HER2^+^	Negative	12 (66.7)	29 (85.3)	40 (78.4)	0.30
	Positive	6 (33.3)	5 (14.7)	11 (21.6)	
Molecular subtype	Luminal A	7 (38.9)	15 (44.1)	28 (54.9)	0.29
	Luminal B	5 (27.8)	2 (5.9)	7 (13.7)	
	HER2+	1 (5.6)	3 (8.8)	4 (7.8)	
	TNBC	5 (27.8)	14 (41.2)	12 (23.5)	
IHP	Good	12 (66.7)	17 (50)	35 (68.6)	0.47
	Moderate	5 (27.8)	14 (41.2)	12 (23.5)	
	Poor	1 (5.6)	3 (8.8)	4 (7.8)	
Recurrence	Negative	11 (61.1)	14 (41.2)	23 (45.1)	0.37
	Positive	7 (38.9)	20 (58.8)	28 (54.9)	
Survival	Alive	7 (38.9)	15 (44.1)	20 (39.2)	0.89
	Dead	11 (61.1)	19 (55.9)	31 (60.8)	

Data are presented as counts (percentage). A two-sided Chi-square test was used. Significance was set at *p* < 0.05.

**Table 5:** Impacts of *DROSHA* and *DICER* mutagenesis in tissue samples with clinicopathological features.

Characteristics	Levels	*DROSHA*			*DICER*		
		No Conversion to A (*n* = 61)	Acquire New A Allele (*n* = 42)	*p*-Value	No Conversion to G (*n* = 59)	Acquire New G Allele (*n* = 44)	*p*-Value
Age	<45 years	29 (47.5)	22 (52.4)	0.69	27 (45.8)	24 (54.5)	0.43
	≥45 years	32 (52.5)	20 (47.6)		32 (54.2)	20 (45.5)	
Affected side	Left	26 (42.6)	12 (28.6)	0.21	23 (39)	15 (34.1)	0.68
	Right	35 (57.4)	30 (71.4)		36 (61)	29 (65.9)	
Number of masses	Single	53 (86.9)	31 (73.8)	0.12	45 (76.3)	39 (88.6)	0.13
	Multiple	8 (13.1)	11 (26.2)		14 (23.7)	5 (11.4)	
Histopathological type	Duct carcinoma	23 (37.7)	16 (38.1)	0.35	23 (39)	16 (36.4)	0.91
	Lobular	11 (18)	12 (28.6)		12 (20.3)	11 (25)	
	Invasive medullary	12 (19.7)	5 (11.9)		11 (18.6)	6 (13.6)	
	Mucinous	10 (16.4)	3 (7.1)		7 (11.9)	6 (13.6)	
	Tubular	2 (3.3)	4 (9.5)		4 (6.8)	2 (4.5)	
	Metaplastic	3 (4.9)	2 (4.8)		2 (3.4)	3 (6.8)	
Grade	G2	45 (73.8)	37 (88.1)	0.09	47 (79.7)	35 (79.5)	0.98
	G3	16 (26.2)	5 (11.9)		12 (20.3)	9 (20.5)	
T stage	T2	31 (50.8)	22 (52.4)	0.26	26 (44.1)	27 (61.4)	0.15
	T3	16 (26.2)	6 (14.3)		13 (22)	9 (20.5)	
	T4	14 (23)	14 (33.3)		20 (33.9)	8 (18.2)	
N stage	N0	12 (19.7)	10 (23.8)	0.63	13 (22)	9 (20.5)	0.85
	N1–3	49 (80.3)	32 (76.2)		46 (78)	35 (79.5)	
M stage	M0	27 (44.3)	20 (47.6)	0.45	29 (49.2)	18 (40.9)	0.71
	M1	28 (45.9)	15 (35.7)		23 (39)	20 (45.5)	
	Mx	6 (9.8)	7 (16.7)		7 (11.9)	6 (13.6)	
LVI	Negative	33 (54.1)	21 (50)	0.69	32 (54.2)	22 (50)	0.67
	Positive	28 (45.9)	21 (50)		27 (45.8)	22 (50)	
Skin infiltration	Negative	53 (86.9)	32 (76.2)	0.19	46 (78)	39 (88.6)	0.16
	Positive	8 (13.1)	10 (23.8)		13 (22)	5 (11.4)	
Clinical stage	IIA	10 (16.4)	6 (14.3)	0.70	8 (13.6)	8 (18.2)	0.68
	IIB	9 (14.8)	6 (14.3)		8 (13.6)	7 (15.9)	
	IIIA	6 (9.8)	7 (16.7)		9 (15.3)	4 (9.1)	
	IIIB	8 (13.1)	8 (19)		11 (18.6)	5 (11.4)	
	IV	28 (45.9)	15 (35.7)		23 (39)	20 (45.5)	
NPI	Good	28 (45.9)	20 (47.6)	0.86	29 (49.2)	19 (43.2)	0.55
	Poor	33 (54.1)	22 (52.4)		30 (50.8)	25 (56.8)	
ESMO	Low risk	23 (37.7)	13 (31)	0.53	21 (35.6)	15 (34.1)	0.87
	High risk	38 (62.3)	29 (69)		38 (64.4)	29 (65.9)	
ER/PR	Positive	43 (70.5)	21 (50)	**0.041**	36 (61)	28 (63.6)	0.83
HER2^+^	Positive	13 (21.3)	9 (21.4)	0.98	12 (20.3)	10 (22.7)	0.81
Molecular subtype	Luminal A	35 (57.4)	15 (35.7)	0.09	27 (45.8)	23 (52.3)	0.48
	Luminal B	8 (13.1)	6 (14.3)		9 (15.3)	5 (11.4)	
	HER2+	5 (8.2)	3 (7.1)		3 (5.1)	5 (11.4)	
	TNBC	13 (21.3)	18 (42.9)		20 (33.9)	11 (25)	
IHP	Good	43 (70.5)	21 (50)	0.06	36 (61)	28 (63.6)	0.37
	Moderate	13 (21.3)	18 (42.9)		20 (33.9)	11 (25)	
	Poor	5 (8.2)	3 (7.1)		3 (5.1)	5 (11.4)	
Recurrence	Negative	29 (47.5)	19 (45.2)	0.84	27 (45.8)	21 (47.7)	0.84
	Positive	32 (52.5)	23 (54.8)		32 (54.2)	23 (52.3)	
Survival	Alive	24 (39.3)	18 (42.9)	0.83	25 (42.4)	17 (38.6)	0.70
	Dead	37 (60.7)	24 (57.1)		34 (57.6)	27 (61.4)	

Data are presented as counts (percentages). LVI: lymphovascular infiltration; ER/PR: estrogen and progesterone receptors; HER2+: HER2/neu receptor, NPI: Nottingham Prognostic Index, calculated as [0.2 × tumor size in cm] + tumor grade [1,2,3] + lymph node stage [1–3, according to stages A–C]; ESMO: European Society of Medical Oncology; IHPI: Immunohistochemical Prognostic Index estimated based on the three-receptor status (HER2, ER, and PR). A two-sided Chi-square test was used. The bold value indicates significance at *p* < 0.05.

**Table 6:** The demographics and comorbidities of the study groups.

Demographic Data		Total (*n* = 212)	Controls (*n* = 106)	Patients (*n* = 106)	*p*-Value
Age	<45 years	103 (48.6)	49 (46.2)	54 (50.9)	0.58
	≥45 years	109 (51.4)	57 (53.8)	52 (49.1)	
Residence	Rural	110 (51.9)	47 (44.3)	63 (59.4)	**0.039**
	Urban	102 (48.1)	59 (55.7)	43 (40.6)	
Marital status	Divorced	44 (20.8)	25 (23.6)	19 (17.9)	0.09
	Married	121 (57.1)	64 (60.4)	57 (53.8)	
	Single	47 (22.2)	17 (16)	30 (28.3)	
Occupation	Housewife	140 (66)	64 (60.4)	76 (71.7)	0.07
	Retired	3 (1.4)	3 (2.8)	0 (0)	
	Worker	69 (32.5)	39 (36.8)	30 (28.3)	
Family history of cancer	Negative	179 (84.4)	106 (100)	73 (68.9)	0.215
	Positive	33 (15.6)	0 (0)	33 (31.1)	
Breast problems	Negative	204 (96.2)	106 (100)	98 (92.5)	**0.007**
	Positive	8 (3.8)	0 (0)	8 (7.5)	
Smoking	Negative	196 (92.5)	101 (95.3)	95 (89.6)	0.192
	Positive	16 (7.5)	5 (4.7)	11 (10.4)	
BMI, Kg/m^2^	Underweight	12 (5.7)	0 (0)	12 (11.3)	0.002
	Normal weight	50 (23.6)	23 (21.7)	27 (25.5)	
	Overweight	65 (30.7)	41 (38.7)	24 (22.6)	
	Obesity	70 (33)	35 (33)	35 (33)	
	Morbid obesity	15 (7.1)	7 (6.6)	8 (7.5)	
Diabetes mellitus	Negative	153 (72.2)	73 (68.9)	80 (75.5)	0.358
	Positive	59 (27.8)	33 (31.1)	26 (24.5)	
Hypertension	Negative	198 (93.4)	99 (93.4)	99 (93.4)	1.000
	Positive	14 (6.6)	7 (6.6)	7 (6.6)	
HCV cirrhosis	Negative	200 (94.3)	98 (92.5)	102 (96.2)	0.374
	Positive	12 (5.7)	8 (7.5)	4 (3.8)	

Data are presented as counts (percentages). A two-sided Chi-square test was used. Bold values indicate significance at *p* < 0.05. BMI: body mass index; HCV: hepatitis C virus.

**Table 7:** Combined genotype association with disease risk.

	*DROSHA*	*DICER*	Total	Controls	Patients	Adjusted OR (95%CI)	*p*-Value
1	G	A	0.4336	0.4311	0.4157	1	---
2	A	G	0.2143	0.134	0.2742	2.18 (1.23–3.89)	**0.008**
3	A	A	0.2008	0.2576	0.1645	0.72 (0.38–1.38)	0.33
4	G	G	0.1513	0.1774	0.1456	1.02 (0.54–1.94)	0.94

Global haplotype association of *DROSHA* rs10719 and *DICER* rs3742330; *p*-value: 0.013. Multivariate regression models were used and are shown as the odds ratio (OR) and 95% confidence interval (95%CI). Adjusted variables were patient age at diagnosis, marital status, occupation, a family history of cancer, prior breast problems, smoking, body mass index, diabetes, hypertension, and hepatitis C virus infection. Bold values indicate significance at *p* < 0.05.

**Table 8:** Multiple regression analysis for the role of *DROSHA* and *DICER* variants in disease outcomes.

Characteristics		*DROSHA*		Adjusted OR (95%CI)	*p*-Value	*DICER*		Adjusted OR (95%CI)	*p*-Value
		A/G-G/G	A/A			A/G-A/A	G/G		
Grade	≤2	57 (81.4)	31 (86.1)	Reference		62 (83.8)	26 (81.3)	Reference	
	>2	13 (18.6)	5 (13.9)	0.55 (0.14–2.07)	0.38	12 (16.2)	6 (18.8)	1.17 (0.32–4.30)	0.80
Masses	Single	51 (72.9)	27 (75)	Reference		56 (75.7)	22 (68.8)	Reference	
	Multiple	19 (27.1)	9 (25.0)	0.61 (0.19–1.94)	0.41	18 (24.3)	10 (31.3)	2.54 (0.77–8.38)	0.12
T stage	≤2	33 (47.1)	19 (52.8)	Reference		37 (50.0)	15 (46.9)	Reference	
	>2	37 (52.9)	17 (47.2)	0.72 (0.27–1.86)	0.49	37 (50.0)	17 (53.1)	1.44 (0.53–3.89)	0.47
N stage	N0	18 (25.7)	13 (36.1)	Reference		20 (27.0)	11 (34.4)	Reference	
	N1-3	52 (74.3)	23 (63.9)	0.68 (0.24–1.86)	0.45	54 (73.0)	21 (65.6)	0.83 (0.28–2.38)	0.72
NPI	Good	33 (47.1)	20 (55.6)	Reference		38 (51.4)	15 (46.9)	Reference	
	Poor	37 (52.9)	16 (44.4)	0.64 (0.24–1.65)	0.35	36 (48.6)	17 (53.1)	1.38 (0.51–3.74)	0.52
LVI	Negative	31 (44.3)	23 (63.9)	Reference		39 (552.7)	15 (46.9)	Reference	
	Positive	39 (55.7)	13 (36.1)	0.33 (0.12–1.09)	0.34	35 (47.3)	17 (53.1)	1.91 (0.66–5.5)	0.22
Skin	Negative	53 (75.7)	30 (83.3)	Reference		57 (77.0)	26 (81.3)	Reference	
	Positive	17 (24.3)	6 (16.7)	0.43 (0.11–1.61)	0.21	17 (23.0)	6 (18.8)	0.96 (0.26–3.52)	0.95

Data are presented as numbers (percentages). Binary logistic regression analysis was applied. NPI: Nottingham Prognostic Index, calculated as [0.2 × tumor size in cm] + tumor grade [1,2,3] + lymph node stage [1–3, according to stages A–C]; LVI: lymphovascular invasion; skin: skin infiltration; OR: odds ratio; CI: confidence interval. Each prognostic outcome represented a separate model. Adjusted variables for each logistic regression model were age, a family history of cancer, prior breast problems, smoking, body mass index, diabetes, hypertension, and hepatitis C virus infection.

## Data Availability

The original contributions presented in this study are included in the article/Supplementary Materials; further inquiries can be directed to the corresponding authors.

## Supplementary Materials

The following supporting information can be downloaded at https://www.mdpi.com/article/10.3390/cimb46090602/s1, Table S1: Bibliography screening of *DROSHA* and *DICER* gene polymorphisms in cancer; Table S2: Characteristics of studies screened for identifying the influence of *DROSHA* and *DICER* genotypes on cancer risk; Table S3: The association between *DROSHA* rs10719 and *DICER* rs3742330 polymorphisms and cancer risk. References [57,58,59,60,61,62,63,64,65] are cited in the Supplementary Materials.

## References

[1] Wilkinson L., Gathani T. (2022). Understanding breast cancer as a global health concern. Br. J. Radiol..

[2] Bray F., Laversanne M., Sung H., Ferlay J., Siegel R.L., Soerjomataram I., Jemal A. (2024). Global cancer statistics 2022: GLOBOCAN estimates of incidence and mortality worldwide for 36 cancers in 185 countries. CA A Cancer J. Clin..

[3] Buocikova V., Rios-Mondragon I., Pilalis E., Chatziioannou A., Miklikova S., Mego M., Pajuste K., Rucins M., Yamani N.E., Longhin E.M. (2020). Epigenetics in Breast Cancer Therapy-New Strategies and Future Nanomedicine Perspectives. Cancers.

[4] Sarvari P., Ramírez-Díaz I., Mahjoubi F., Rubio K. (2022). Advances of Epigenetic Biomarkers and Epigenome Editing for Early Diagnosis in Breast Cancer. Int. J. Mol. Sci..

[5] Plawgo K., Raczynska K.D. (2022). Context-Dependent Regulation of Gene Expression by Non-Canonical Small RNAs. Noncoding RNA.

[6] Eastlack S.C., Alahari S.K. (2015). MicroRNA and Breast Cancer: Understanding Pathogenesis, Improving Management. Noncoding RNA.

[7] Toraih E.A., Mohammed E.A., Farrag S., Ramsis N., Hosny S. (2015). Pilot Study of Serum MicroRNA-21 as a Diagnostic and Prognostic Biomarker in Egyptian Breast Cancer Patients. Mol. Diagn. Ther..

[8] O’Brien J., Hayder H., Zayed Y., Peng C. (2018). Overview of MicroRNA Biogenesis, Mechanisms of Actions, and Circulation. Front. Endocrinol..

[9] Flores-Jasso C.F., Arenas-Huertero C., Reyes J.L., Contreras-Cubas C., Covarrubias A., Vaca L. (2009). First step in pre-miRNAs processing by human Dicer. Acta Pharmacol. Sin..

[10] Yan M., Huang H.Y., Wang T., Wan Y., Cui S.D., Liu Z.Z., Fan Q.X. (2012). Dysregulated expression of dicer and drosha in breast cancer. Pathol. Oncol. Res. POR.

[11] Avery-Kiejda K.A., Braye S.G., Forbes J.F., Scott R.J. (2014). The expression of Dicer and Drosha in matched normal tissues, tumours and lymph node metastases in triple negative breast cancer. BMC Cancer.

[12] Otmani K., Lewalle P. (2021). Tumor Suppressor miRNA in Cancer Cells and the Tumor Microenvironment: Mechanism of Deregulation and Clinical Implications. Front. Oncol..

[13] Ali Syeda Z., Langden S.S.S., Munkhzul C., Lee M., Song S.J. (2020). Regulatory Mechanism of MicroRNA Expression in Cancer. Int. J. Mol. Sci..

[14] Machowska M., Galka-Marciniak P., Kozlowski P. (2022). Consequences of genetic variants in miRNA genes. Comput. Struct. Biotechnol. J..

[15] Elshazli R.M., Toraih E.A., Hussein M.H., Ruiz E.M., Kandil E., Fawzy M.S. (2023). Pan-Cancer Study on Variants of Canonical miRNA Biogenesis Pathway Components: A Pooled Analysis. Cancers.

[16] Jiang Y., Chen J., Wu J., Hu Z., Qin Z., Liu X., Guan X., Wang Y., Han J., Jiang T. (2013). Evaluation of genetic variants in microRNA biosynthesis genes and risk of breast cancer in Chinese women. Int. J. Cancer.

[17] Kamalabad S.T., Zamanzadeh Z., Rezaei H., Tabatabaeian M., Abkar M. (2023). Association of DROSHA rs6877842, rs642321 and rs10719 polymorphisms with increased susceptibility to breast cancer: A case-control study with genotype and haplotype analysis. Breast Dis..

[18] Quan C., Ping J., Lu H., Zhou G., Lu Y. (2022). 3DSNP 2.0: Update and expansion of the noncoding genomic variant annotation database. Nucleic Acids Res..

[19] Kurshumliu F., Gashi-Luci L., Kadare S., Alimehmeti M., Gozalan U. (2014). Classification of patients with breast cancer according to Nottingham prognostic index highlights significant differences in immunohistochemical marker expression. World J. Surg. Oncol..

[20] Loibl S., André F., Bachelot T., Barrios C.H., Bergh J., Burstein H.J., Cardoso M.J., Carey L.A., Dawood S., Del Mastro L. (2024). Early breast cancer: ESMO Clinical Practice Guideline for diagnosis, treatment and follow-up. Ann. Oncol..

[21] Toraih E.A., Fawzy M.S., Mohammed E.A., Hussein M.H., El-Labban M.M. (2016). MicroRNA-196a2 Biomarker and Targetome Network Analysis in Solid Tumors. Mol. Diagn. Ther..

[22] Al Ageeli E., Attallah S.M., Mohamed M.H., Almars A.I., Kattan S.W., Toraih E.A., Fawzy M.S., Darwish M.K. (2022). Migration/Differentiation-Associated LncRNA SENCR rs12420823*C/T: A Novel Gene Variant Can Predict Survival and Recurrence in Patients with Breast Cancer. Genes.

[23] Fawzy M.S., Ibrahiem A.T., Osman D.M., Almars A.I., Alshammari M.S., Almazyad L.T., Almatrafi N.D.A., Almazyad R.T., Toraih E.A. (2024). Angio-Long Noncoding RNA MALAT1 (rs3200401) and MIAT (rs1061540) Gene Variants in Ovarian Cancer. Epigenomes.

[24] Amer M.H. (2014). Genetic factors and breast cancer laterality. Cancer Manag. Res..

[25] Barbara R.C., Piotr R., Kornel B., Elzbieta Z., Danuta R., Eduardo N. (2020). Divergent Impact of Breast Cancer Laterality on Clinicopathological, Angiogenic, and Hemostatic Profiles: A Potential Role of Tumor Localization in Future Outcomes. J. Clin. Med..

[26] Hynes C., Kakumani P.K. (2024). Regulatory role of RNA-binding proteins in microRNA biogenesis. Front. Mol. Biosci..

[27] Gu K., Mok L., Wakefield M.J., Chong M.M.W. (2024). Non-canonical RNA substrates of Drosha lack many of the conserved features found in primary microRNA stem-loops. Sci. Rep..

[28] Song M.S., Rossi J.J. (2017). Molecular mechanisms of Dicer: Endonuclease and enzymatic activity. Biochem. J..

[29] Theotoki E.I., Pantazopoulou V.I., Georgiou S., Kakoulidis P., Filippa V., Stravopodis D.J., Anastasiadou E. (2020). Dicing the Disease with Dicer: The Implications of Dicer Ribonuclease in Human Pathologies. Int. J. Mol. Sci..

[30] Yousefnia S., Negahdary M. (2024). Role of miRNAs in Cancer: Oncogenic and Tumor Suppressor miRNAs, Their Regulation and Therapeutic Applications.

[31] Cho S.H., Ko J.J., Kim J.O., Jeon Y.J., Yoo J.K., Oh J., Oh D., Kim J.W., Kim N.K. (2015). 3′-UTR Polymorphisms in the MiRNA Machinery Genes DROSHA, DICER1, RAN, and XPO5 Are Associated with Colorectal Cancer Risk in a Korean Population. PLoS ONE.

[32] Yuan L., Chu H., Wang M., Gu X., Shi D., Ma L., Zhong D., Du M., Li P., Tong N. (2013). Genetic variation in DROSHA 3'UTR regulated by hsa-miR-27b is associated with bladder cancer risk. PLoS ONE.

[33] Kim J., Lee J., Oh J.H., Chang H.J., Sohn D.K., Kwon O., Shin A., Kim J. (2019). Dietary Lutein Plus Zeaxanthin Intake and DICER1 rs3742330 A > G Polymorphism Relative to Colorectal Cancer Risk. Sci. Rep..

[34] Song X., Zhong H., Wu Q., Wang M., Zhou J., Zhou Y., Lu X., Ying B. (2017). Association between SNPs in microRNA machinery genes and gastric cancer susceptibility, invasion, and metastasis in Chinese Han population. Oncotarget.

[35] Liao Y., Liao Y., Li J., Liu L., Li J., Wan Y., Peng L. (2018). Genetic variants in miRNA machinery genes associated with clinicopathological characteristics and outcomes of gastric cancer patients. Int. J. Biol. Markers.

[36] Kim M.N., Kim J.O., Lee S.M., Park H., Lee J.H., Rim K.S., Hwang S.G., Kim N.K. (2016). Variation in the Dicer and RAN Genes Are Associated with Survival in Patients with Hepatocellular Carcinoma. PLoS ONE.

[37] Nikolic Z., Savic Pavicevic D., Vucic N., Cerovic S., Vukotic V., Brajuskovic G. (2017). Genetic variants in RNA-induced silencing complex genes and prostate cancer. World J. Urol..

[38] Osuch-Wojcikiewicz E., Bruzgielewicz A., Niemczyk K., Sieniawska-Buccella O., Nowak A., Walczak A., Majsterek I. (2015). Association of Polymorphic Variants of miRNA Processing Genes with Larynx Cancer Risk in a Polish Population. BioMed Res. Int..

[39] Mohammadpour-Gharehbagh A., Heidari Z., Eskandari M., Aryan A., Salimi S. (2020). Association between Genetic Polymorphisms in microRNA Machinery Genes and Risk of Papillary Thyroid Carcinoma. Pathol. Oncol. Res. POR.

[40] Huang S.Q., Zhou Z.X., Zheng S.L., Liu D.D., Ye X.H., Zeng C.L., Han Y.J., Wen Z.H., Zou X.Q., Wu J. (2018). Association of variants of miRNA processing genes with cervical precancerous lesion risk in a southern Chinese population. Biosci. Rep..

[41] Han Y., Liu Y., Gui Y., Cai Z. (2013). Inducing cell proliferation inhibition and apoptosis via silencing Dicer, Drosha, and Exportin 5 in urothelial carcinoma of the bladder. J. Surg. Oncol..

[42] Cardoso J.V., Medeiros R., Dias F., Costa I.A., Ferrari R., Berardo P.T., Perini J.A. (2021). DROSHA rs10719 and DICER1 rs3742330 polymorphisms in endometriosis and different diseases: Case-control and review studies. Exp. Mol. Pathol..

[43] Hannafon B.N., Cai A., Calloway C.L., Xu Y.F., Zhang R., Fung K.M., Ding W.Q. (2019). miR-23b and miR-27b are oncogenic microRNAs in breast cancer: Evidence from a CRISPR/Cas9 deletion study. BMC Cancer.

[44] Cheng H., Li H., Feng Y., Zhang Z. (2018). Correlation analysis between SNPs in microRNA-machinery genes and tuberculosis susceptibility in the Chinese Uygur population. Medicine.

[45] Mitra A., Rostas J.W., Dyess D.L., Shevde L.A., Samant R.S. (2012). Micro-RNA-632 downregulates DNAJB6 in breast cancer. Lab. Investig. A J. Tech. Methods Pathol..

[46] Zheng A., Song X., Zhang L., Zhao L., Mao X., Wei M., Jin F. (2019). Long noncoding RNA LUCAT1/miR-5582-3p/TCF7L2 axis regulates breast cancer stemness via Wnt/beta-catenin pathway. J. Exp. Clin. Cancer Res. CR.

[47] Madu C.O., Wang S., Madu C.O., Lu Y. (2020). Angiogenesis in Breast Cancer Progression, Diagnosis, and Treatment. J. Cancer.

[48] Iorio M.V., Croce C.M. (2012). microRNA involvement in human cancer. Carcinogenesis.

[49] Shin H.T., Choi Y.L., Yun J.W., Kim N.K.D., Kim S.Y., Jeon H.J., Nam J.Y., Lee C., Ryu D., Kim S.C. (2017). Prevalence and detection of low-allele-fraction variants in clinical cancer samples. Nat. Commun..

[50] Sharma A., Merritt E., Hu X., Cruz A., Jiang C., Sarkodie H., Zhou Z., Malhotra J., Riedlinger G.M., De S. (2019). Non-Genetic Intra-Tumor Heterogeneity Is a Major Predictor of Phenotypic Heterogeneity and Ongoing Evolutionary Dynamics in Lung Tumors. Cell Rep..

[51] Solis-Moruno M., Batlle-Maso L., Bonet N., Arostegui J.I., Casals F. (2023). Somatic genetic variation in healthy tissue and non-cancer diseases. Eur. J. Hum. Genet. EJHG.

[52] Zhu J.W., Charkhchi P., Adekunte S., Akbari M.R. (2023). What Is Known about Breast Cancer in Young Women?. Cancers.

[53] Yap Y.S. (2023). Outcomes in breast cancer-does ethnicity matter?. ESMO Open.

[54] Feng Y., Spezia M., Huang S., Yuan C., Zeng Z., Zhang L., Ji X., Liu W., Huang B., Luo W. (2018). Breast cancer development and progression: Risk factors, cancer stem cells, signaling pathways, genomics, and molecular pathogenesis. Genes Dis..

[55] Lim I.Y., Lin X., Karnani N., Patel V., Preedy V. (2017). Implications of Genotype and Environment on Variation in DNA Methylation. Handbook of Nutrition, Diet, and Epigenetics.

[56] Santalo J., Berdasco M. (2022). Ethical implications of epigenetics in the era of personalized medicine. Clin. Epigenetics.

[57] Bermisheva M.A., Takhirova Z.R., Gilyazova I.R., Khusnutdinova E.K. (2018). MicroRNA Biogenesis Pathway Gene Polymorphisms Are Associated with Breast Cancer Risk. Russian J. Genet..

[58] Martin-Guerrero I., Gutierrez-Camino A., Lopez-Lopez E., Bilbao-Aldaiturriaga N., Pombar-Gomez M., Ardanaz M., Garcia-Orad A. (2015). Genetic variants in miRNA processing genes and pre-miRNAs are associated with the risk of chronic lymphocytic leukemia. PLoS ONE.

[59] Kim J.S., Choi Y.Y., Jin G., Kang H.G., Choi J.E., Jeon H.S., Lee W.K., Kim D.S., Kim C.H., Kim Y.J. (2010). Association of a common AGO1 variant with lung cancer risk: A two-stage case-control study. Mol. Carcinog..

[60] Yang H., Dinney C.P., Ye Y., Zhu Y., Grossman H.B., Wu X. (2008). Evaluation of genetic variants in microRNA-related genes and risk of bladder cancer. Cancer Res..

[61] Horikawa Y., Wood C.G., Yang H., Zhao H., Ye Y., Gu J., Lin J., Habuchi T., Wu X. (2008). Single nucleotide polymorphisms of microRNA machinery genes modify the risk of renal cell carcinoma. Clin. Cancer Res..

[62] Oz M., Karakus S., Yildirim M., Bagci B., Sari I., Bagci G., Yildiz C., Akkar O., Cetin A., Yanik A. (2018). Genetic variants in the microRNA machinery gene (Dicer) have a prognostic value in the management of endometrial cancer. J. Cancer Res. Ther..

[63] Yuan W.W., Hang D., Wang L.H., Chen S.H., Ding Z.X., Hu Z.B., Ma H.X. (2016). Association between genetic variants in microRNA biosynthesis genes and the risk of head and neck squamous cell carcinoma. Zhonghua Liu Xing Bing Xue Za Zhi.

[64] Peckham-Gregory E.C., Thapa D.R., Martinson J., Duggal P., Penugonda S., Bream J.H., Chang P.Y., Dandekar S., Chang S.C., Detels R. (2016). MicroRNA-related polymorphisms and non-Hodgkin lymphoma susceptibility in the Multicenter AIDS Cohort Study. Cancer Epidemiol..

[65] Zheng L., Gu H., Zhang L., Wang Z. (2013). DICER rs3742330 A>G polymorphism and risk of esophageal cancer. Chin. J. Cancer Prev. Treat..

