# Supplementary material for: Impacts of DROSHA (rs10719) and DICER (rs3742330) Variants on Breast Cancer Risk and Their Distribution in Blood and Tissue Samples of Egyptian Patients
Source: Curr Issues Mol Biol. 2024 Sep 12;46(9):10087–111. doi: 10.3390/cimb46090602 (PMC11430749; doi:10.3390/cimb46090602)
Supplement: Supplementary file 1 [file cimb-46-00602-s001.zip › cimb-3195228-supplementary.pdf]

Supplementary Table S1. Bibliography screening of *DROSHA* and *DICER* gene polymorphisms in cancer.

Gene	SNP ID	Alleles	MAF	Ancestral	Location	dbSNP	Position	Total Citations	Citations for cancer
DROSHA	**rs10719**	A/G	0.48 (A)	A	31401340	A>G	3' UTR	66	10
	rs6877842	G/C	0.14 (C)	G	31532531	G>C	Intron	25	8
	rs642321	T/C	0.32 (T)	T	31400896	T>C	3' UTR	17	3
	rs644236	T/C	0.47 (C)	C	31409008	T>C	Intron	7	1
	rs2287584	T/C	0.45 (C)	C	31422900	T>C	Synonymous	4	1
	rs2291109	A/G/T	0.06 (A)	T	31532322	A>G / A>T	5' UTR	2	1
	rs3805500	G/A	0.50 (G)	G	31462870	G>A	Intron	6	1
	rs4867329	A/C	0.37 (C)	A	31435520	A>C	Intron	6	1
DICER	**rs3742330**	A/G	0.14 (G)	A	95087025	A>G	3' UTR	71	16
	rs13078	A/T	0.09 (A)	T	95090410	A>T	3' UTR	39	9
	rs1057035	T/C	0.17 (C)	T	95087805	T>C	3' UTR	35	11
	rs12323635	C/T	0.49 (T)	T	95159374	C>T	Intron	8	1

Supplementary Table S2. Characteristics of studies screened for identifying the role of *DROSHA* and *DICER* genotypes on cancer risk.

First author, year [Ref]	Country	Cancer site	Cases	Controls	Genotyping method
***DROSHA* rs10719 A > G**					
Bermisheva, 2018 [1]	Russia	Breast	413	361	TaqMan OpenArray Genotyping
Song, 2017 [2]	China	Gastric	628	502	HRMA
Kim, 2016 [3]	South Korea	Liver	147	209	PCR-RFLP
Martin-Guerrero, 2015 [4]	Spain	CLL	102	345	TaqMan OpenArray Genotyping
Cho, 2015 [5]	South Korea	Colorectal	408	400	PCR-RFLP
Yuan, 2013 [6]	China	Bladder	684	727	TaqMan genotyping assay
Jiang, 2013 [7]	China	Breast	847	878	TaqMan OpenArray Genotyping
Kim, 2010 [8]	Republic of Korea	Lung	97	97	SMSG
Yang, 2008 [9]	USA	Bladder	534	442	SNPlex technology
Horikawa, 2008 [10]	USA	Renal	252	246	SNPlex technology
***DICER* rs3742330 A > G**					
Mohammadpour-Gharehbagh, 2020 [11]	Iran	Thyroid	120	130	PCR-RFLP
Kim, 2019 [12]	South Korea	Colorectal	700	1400	MassARRAY iPLEX Gold Assay
Oz, 2018 [13]	Turkey	Endometrium	80	79	TaqMan genotyping assay
Huang, 2018 [14]	China	Cervix	296	296	MALDI-TOF MS method
Song, 2017 [2]	China	Gastric	628	502	HRMA
Nikolic, 2017 [15]	Serbia	Prostate	353	318	HRMA
Yuan, 2016 [16]	China	Head& Neck	575	1551	Illumina Infinium BeadChip platform
Peckham-Gregory, 2016 [17]	USA	NHL	180	529	Customized Fluidigm Dynamic 96.96 ArrayTM assay
Kim, 2016 [3]	South Korea	Liver	147	209	PCR-RFLP
Osuch-Wojcikiewicz, 2015 [18]	Poland	Larynx	123	170	TaqMan genotyping assay
Cho, 2015 [5]	South Korea	CRC	408	400	PCR-RFLP
Zheng, 2013 [19]	China	Esophagus	380	380	MALDI-TOF MS method
Yuan, 2013 [6]	China	Bladder	683	727	TaqMan genotyping assay
Kim, 2010 [8]	Republic of Korea	Lung	100	100	SMSG
Yang, 2008 [9]	USA	Bladder	727	724	SNPlex technology
Horikawa, 2008 [10]	USA	Renal	277	278	SNPlex technology

CLL: chronic lymphocytic leukemia, NHL: Non-Hodgkin lymphoma.

Supplementary Table S3. The association between DROSHA*rs10719 and DICER*rs3742330 polymorphisms and cancer risk.

Cancer type	No. of studies	Sample size		Test of association			
		Cancer	Control	OR	95% CI	*P*-value	Model
***DROSHA**rs10719**							
Overall	10	8224	8414	1.032	0.958-1.113	0.40	R
Bladder cancer	2	2436	2338	1.039	0.740-1.460	0.82	R
Breast cancer	2	2520	2478	1.198	0.927-1.549	0.16	R
CLL	1	204	690	0.655	0.440-0.977	**0.038**	F
Colorectal cancer	1	816	800	0.999	0.800-1.247	0.99	F
Gastric cancer	1	1256	1004	0.970	0.809-1.163	0.74	F
HCC	1	294	418	1.029	0.734-1.442	0.86	F
Lung Cancer	1	194	194	0.873	0.553-1.379	0.56	F
Renal cell carcinoma	1	504	492	0.981	0.725-1.329	0.90	F
***DICER**rs3742330**							
Overall	16	11554	15586	0.974	0.889-1.066	0.56	R
Colorectal cancer	2	2216	3600	1.002	0.822-1.220	0.98	R
Bladder cancer	2	2820	2902	1.034	0.905-1.182	0.61	F
PTC	1	240	260	0.553	0.342-0.894	0.01	F
Endometrial cancer	1	160	158	0.626	0.333-1.174	0.14	F
Cervical precancerous lesions	1	592	592	0.730	0.578-0.924	**0.009**	F
Gastric cancer	1	1256	1004	0.762	0.642-0.905	**0.002**	F
Prostate cancer	1	706	636	1.029	0.723-1.462	0.87	F
Head/neck carcinoma	1	1150	3102	0.998	0.867-1.149	0.97	F
NHL	1	360	1058	0.883	0.580-1.345	0.56	F
HCC	1	294	418	1.020	0.755-1.378	0.90	F
Larynx cancer	1	246	340	1.413	1.005-1.987	**0.047**	F
Oesophageal cancer	1	760	760	1.117	0.909-1.373	0.29	F
Lung cancer	1	200	200	1.359	0.914-2.023	0.13	F
Renal cell carcinoma	1	554	556	0.863	0.555-1.341	0.51	F

The allelic model (G allele versus A allele) was employed. OR: odds ratio, CI: confidence interval, F: fixed-effects model, R: Random-effects model. CLL: chronic lymphocytic leukemia, HCC: hepatocellular carcinoma, PTC: papillary thyroid cancer, NHL: non-Hodgkin lymphoma. Bold values indicate significance at p-value < 0.05.

References of Suppl. Table S2

[1] Bermisheva, M.A.; Takhirova, Z.R.; Gilyazova, I.R.; Khusnutdinova, E.K. MicroRNA Biogenesis Pathway Gene Polymorphisms Are Associated with Breast Cancer Risk. Russian Journal of Genetics 2018, 54, 568-575, doi:10.1134/s1022795418040051.

[2] Song, X.; Zhong, H.; Wu, Q.; Wang, M.; Zhou, J.; Zhou, Y.; Lu, X.; Ying, B. Association between SNPs in microRNA machinery genes and gastric cancer susceptibility, invasion, and metastasis in Chinese Han population. Oncotarget 2017, 8, 86435-86446, doi:10.18632/oncotarget.21199.

[3] Kim, M.N.; Kim, J.O.; Lee, S.M.; Park, H.; Lee, J.H.; Rim, K.S.; Hwang, S.G.; Kim, N.K. Variation in the Dicer and RAN Genes Are Associated with Survival in Patients with Hepatocellular Carcinoma. PLoS One 2016, 11, e0162279, doi:10.1371/journal.pone.0162279.

[4] Martin-Guerrero, I.; Gutierrez-Camino, A.; Lopez-Lopez, E.; Bilbao-Aldaiturriaga, N.; Pombar-Gomez, M.; Ardanaz, M.; Garcia-Orad, A. Genetic variants in miRNA processing genes and pre-miRNAs are associated with the risk of chronic lymphocytic leukemia. PLoS One 2015, 10, e0118905, doi:10.1371/journal.pone.0118905.

[5] Cho, S.H.; Ko, J.J.; Kim, J.O.; Jeon, Y.J.; Yoo, J.K.; Oh, J.; Oh, D.; Kim, J.W.; Kim, N.K. 3'-UTR Polymorphisms in the MiRNA Machinery Genes DROSHA, DICER1, RAN, and XPO5 Are Associated with Colorectal Cancer Risk in a Korean Population. PLoS One 2015, 10, e0131125, doi:10.1371/journal.pone.0131125.

[6] Yuan, L.; Chu, H.; Wang, M.; Gu, X.; Shi, D.; Ma, L.; Zhong, D.; Du, M.; Li, P.; Tong, N.; et al. Genetic variation in DROSHA 3'UTR regulated by hsa-miR-27b is associated with bladder cancer risk. PLoS One 2013, 8, e81524, doi:10.1371/journal.pone.0081524.

[7] Jiang, Y.; Chen, J.; Wu, J.; Hu, Z.; Qin, Z.; Liu, X.; Guan, X.; Wang, Y.; Han, J.; Jiang, T.; et al. Evaluation of genetic variants in microRNA biosynthesis genes and risk of breast cancer in Chinese women. International journal of cancer 2013, 133, 2216-2224, doi:10.1002/ijc.28237.

[8] Kim, J.S.; Choi, Y.Y.; Jin, G.; Kang, H.G.; Choi, J.E.; Jeon, H.S.; Lee, W.K.; Kim, D.S.; Kim, C.H.; Kim, Y.J.; et al. Association of a common AGO1 variant with lung cancer risk: a two-stage case-control study. Mol Carcinog 2010, 49, 913-921, doi:10.1002/mc.20672.

[9] Yang, H.; Dinney, C.P.; Ye, Y.; Zhu, Y.; Grossman, H.B.; Wu, X. Evaluation of genetic variants in microRNA-related genes and risk of bladder cancer. Cancer Res 2008, 68, 2530-2537, doi:10.1158/0008-5472.Can-07-5991.

[10] Horikawa, Y.; Wood, C.G.; Yang, H.; Zhao, H.; Ye, Y.; Gu, J.; Lin, J.; Habuchi, T.; Wu, X. Single nucleotide polymorphisms of microRNA machinery genes modify the risk of renal cell carcinoma. Clin Cancer Res 2008, 14, 7956-7962, doi:10.1158/1078-0432.CCR-08-1199.

[11] Mohammadpour-Gharehbagh, A.; Heidari, Z.; Eskandari, M.; Aryan, A.; Salimi, S. Association between Genetic Polymorphisms in microRNA Machinery Genes and Risk of Papillary Thyroid Carcinoma. Pathol Oncol Res 2020, 26, 1235-1241, doi:10.1007/s12253-019-00688-z.

[12] Kim, J.; Lee, J.; Oh, J.H.; Chang, H.J.; Sohn, D.K.; Kwon, O.; Shin, A.; Kim, J. Dietary Lutein Plus Zeaxanthin Intake and DICER1 rs3742330 A > G Polymorphism Relative to Colorectal Cancer Risk. Sci Rep 2019, 9, 3406, doi:10.1038/s41598-019-39747-5.

[13] Oz, M.; Karakus, S.; Yildirim, M.; Bagci, B.; Sari, I.; Bagci, G.; Yildiz, C.; Akkar, O.; Cetin, A.; Yanik, A. Genetic variants in the microRNA machinery gene (Dicer) have a prognostic value in the management of endometrial cancer. J Cancer Res Ther 2018, 14, 1279-1284, doi:10.4103/0973-1482.187291.

[14] Huang, S.Q.; Zhou, Z.X.; Zheng, S.L.; Liu, D.D.; Ye, X.H.; Zeng, C.L.; Han, Y.J.; Wen, Z.H.; Zou, X.Q.; Wu, J.; et al. Association of variants of miRNA processing genes with cervical precancerous lesion risk in a southern Chinese population. Biosci Rep 2018, 38, doi:10.1042/BSR20171565.

[15] Nikolic, Z.; Savic Pavicevic, D.; Vucic, N.; Cerovic, S.; Vukotic, V.; Brajuskovic, G. Genetic variants in RNA-induced silencing complex genes and prostate cancer. World J Urol 2017, 35, 613-624, doi:10.1007/s00345-016-1917-0.

[16] Yuan, W.W.; Hang, D.; Wang, L.H.; Chen, S.H.; Ding, Z.X.; Hu, Z.B.; Ma, H.X. [Association between genetic variants in microRNA biosynthesis genes and the risk of head and neck squamous cell carcinoma]. Zhonghua Liu Xing Bing Xue Za Zhi 2016, 37, 1069-1073, doi:10.3760/cma.j.issn.0254-6450.2016.08.003.

[17] Peckham-Gregory, E.C.; Thapa, D.R.; Martinson, J.; Duggal, P.; Penugonda, S.; Bream, J.H.; Chang, P.Y.; Dandekar, S.; Chang, S.C.; Detels, R.; et al. MicroRNA-related polymorphisms and non-Hodgkin lymphoma susceptibility in the Multicenter AIDS Cohort Study. Cancer Epidemiol 2016, 45, 47-57, doi:10.1016/j.canep.2016.09.007.

[18] Osuch-Wojcikiewicz, E.; Bruzgielewicz, A.; Niemczyk, K.; Sieniawska-Buccella, O.; Nowak, A.; Walczak, A.; Majsterek, I. Association of Polymorphic Variants of miRNA Processing Genes with Larynx Cancer Risk in a Polish Population. Biomed Res Int 2015, 2015, 298378, doi:10.1155/2015/298378.

[19] Zheng, L.; Gu, H.; Zhang, L.; Wang, Z. DICER rs3742330 A>G polymorphism and risk of esophageal cancer. Chinese Journal of Cancer Prevention and Treatment 2013, 20, 1794-1796.
